# Annealed Importance Sampling for Neural Mass Models

**DOI:** 10.1371/journal.pcbi.1004797

**Published:** 2016-03-04

**Authors:** Will Penny, Biswa Sengupta

**Affiliations:** Wellcome Trust Centre for Neuroimaging, University College London, London, United Kingdom; Brain and Spine Institute (ICM), FRANCE

## Abstract

Neural Mass Models provide a compact description of the dynamical activity of cell populations in neocortical regions. Moreover, models of regional activity can be connected together into networks, and inferences made about the strength of connections, using M/EEG data and Bayesian inference. To date, however, Bayesian methods have been largely restricted to the Variational Laplace (VL) algorithm which assumes that the posterior distribution is Gaussian and finds model parameters that are only locally optimal. This paper explores the use of Annealed Importance Sampling (AIS) to address these restrictions. We implement AIS using proposals derived from Langevin Monte Carlo (LMC) which uses local gradient and curvature information for efficient exploration of parameter space. In terms of the estimation of Bayes factors, VL and AIS agree about which model is best but report different degrees of belief. Additionally, AIS finds better model parameters and we find evidence of non-Gaussianity in their posterior distribution.

## Author Summary

The activity of populations of neurons in the human brain can be described using a set of differential equations known as a neural mass model. These models can then be connected to describe activity in multiple brain regions and, by fitting them to human brain imaging data, statistical inferences can be made about changes in macroscopic connectivity among brain regions. For example, the strength of a connection from one region to another may be more strongly engaged in a particular patient population or during a specific cognitive task. Current statistical inference approaches use a Bayesian algorithm based on principles of local optimization and the assumption that uncertainty about model parameters (e.g. connectivity), having seen the data, follows a Gaussian distribution. This paper evaluates current methods against a global Bayesian optimization algorithm and finds that the two approaches (local/global) agree about which model is best, but finds that the global approach produces better parameter estimates.

## Introduction

Dynamical systems models instantiated using differential equations are a mainstay of modern neuroscience and provide mathematical descriptions of neuronal activity over multiple spatial and temporal scales [1, 2]. In imaging neuroscience a widely adopted framework, called Dynamic Causal Modelling (DCM), has been developed for fitting such models to brain imaging data using a Bayesian approach [3]. This allows inferences to be made about changes in parameters (eg. effective connectivity) in the human brain using noninvasive imaging data. There is now a library of DCMs which differ according to their level of biological realism and the data features they explain. DCM can be applied to fMRI [3], EEG and MEG [4] and invasive electrophysiological data [5].

The Bayesian approach to model fitting in DCM is based on the Variational Laplace (VL) algorithm [6]. One of its core assumptions, the ‘Laplace Assumption’, is that the posterior distribution is Gaussian. This assumption is typically instantiated by finding the maximum posterior parameter vector, using numerical optimisation, and making a Taylor expansion around this value and retaining terms up to second order [7]. It has been found to be more robust than higher-order moment expansions on empirical data [8]. In VL, the posterior is assumed to factorise into a product of probability distributions, one over latent variables controlling noise variances and one over model parameters. Each distribution is multivariate Gaussian with mean and covariance that are iteratively updated to maximise an approximation to the model evidence [6].

The Laplace approximation is attractive because it provides a computationally simple method for both quantifying posterior uncertainty in model parameters and approximating the model evidence for Bayesian model comparison.

A theoretical motivation for the the Laplace approximation is that the posterior will tend to a Gaussian in the limit where the number of data points goes to infinity [9]. But as previously noted in the context of DCM [10], it is questionable as to whether posteriors are Gaussian for datasets that are encountered in practice which naturally have a finite number of data points. The VL algorithm has two potential weaknesses (i) as with any local optimisation method working in a non-convex domain [11] it may fall into a local maxima and (ii) the distribution around the maxima may be non-Gaussian.

In this paper we compare VL to Monte Carlo methods in the challenging context of identifying Neural Mass Models (NMMs) [12]. The advantage of Monte Carlo methods is that, provided the sampling process runs for a sufficiently long time, the samples converge in distribution to the exact posterior. This obviates the need for Gaussian assumptions but at the cost of potentially very long sampling times. To address these issues this paper uses the Annealed Importance Sampling (AIS) algorithm [13] with proposals made using a Langevin Monte Carlo (LMC) procedure [14]. The use of AIS has two benefits (i) it can accomodate multiple local maxima and (ii) it provides an estimate of the Bayesian model evidence. The use of LMC improves convergence properties because proposals are made using local gradient and curvature information [14, 15].

Previously, the Metropolis-Hastings (MH) algorithm has been used to validate VL in the context of DCM for fMRI [16]. Whilst these findings are largely consistent with the Laplace assumption this study is incomplete in a number of respects (i) only results from a single Markov chain were reported thus raising the possibility that a local maxima was found, (ii) no sample-based estimate of the model evidence was provided, and (iii) the neurodynamical models used in fMRI are based on linear dynamical systems, so this finding may not hold for the nonlinear dynamical models [17] underlying other DCMs such as those for M/EEG data.

This paper assesses how well the two Bayesian estimation algorithms (AIS-LMC and VL) perform inference for NMMs. These models have been chosen as they are highly nonlinear and underlie the first proposed DCM for M/EEG data [17]. In order to validate our software implementation and fine tune parameters of the AIS algorithm, we additionally evaluate these algorithms in the simpler context of linear and nonlinear regression models.

## Materials and Methods

In what follows N(x;m,Λ) denotes a multivariate Gaussian variable *x* with mean *m* and precision Λ. We consider Bayesian inference for data *Y*, or *y*, models with parameters *w*, priors *p*(*w*) and likelihoods *p*(*Y*|*w*) or *p*(*y*|*w*). All models in this paper use Gaussian priors with mean *μ* and precision Λ. In the subsections that follow we describe the AIS algorithm and show how LMC can be used within it to provide proposals. We then describe the linear regression, nonlinear regression and neural mass models that we will use to test the inference methods. To provide a convenient reference for some of the underlying concepts we provide supplementary material on Importance Sampling S1 Text, Fisher Information S2 Text, Neural Mass Models S3 Text, Variational Laplace S4 Text and Chib’s method for estimating model evidence S5 Text.

### Annealed Importance Sampling

Annealed Importance Sampling (AIS) [13] provides samples from a posterior density using a sequence of densities at a series of monotonically increasing inverse temperatures *β*_*j*_ with *j* = 0..*J*, *β*_0_ = 0 and *β*_*J*_ = 1. For the *j*th temperature the algorithm produces a sample from the unnormalised density

$$f_{j}\left(w\right)=p{\left(y|w\right)}^{\beta_{j}}p\left(w\right)$$ (1)

An independent sample *w*^(*i*)^ from the posterior density is produced by generating a sequence of points *w*_1_, *w*_2_, … *w*_*J*_ as follows

- Generate *w*_1_ from *p*(*w*)
- Generate *w*_2_ from *w*_1_ using *T*_1_(*w*_2_|*w*_1_)
- …
- Generate *w*_*j*_ from *w*_*j*−1_ using *T*_*j*−1_(*w*_*j*_|*w*_*j*−1_)
- …
- Generate *w*_*J*_ from *w*_*J*−1_ using *T*_*J*−1_(*w*_*J*_|*w*_*J*−1_)

and then let *w*^(*i*)^ = *w*_*J*_. We refer to the process of producing a single independent sample as a ‘trajectory’. The transition densities *T*_*j*_ can be chosen in any of the usual ways for constructing Markov chains [18] and may themselves involve several steps. The only requirement is that *T*_*j*_ is chosen to leave *f*_*j*_ as the invariant distribution. For example, for a simple density estimation problem, Neal [13] specified each *T*_*j*_ to be a sequence of Metropolis moves each defined using an isotropic Gaussian proposal with increasing width. For a linear regression problem with non-Gaussian priors he employed a Hamiltonian Monte-Carlo (HMC) approach [19]. In this paper we will use Langevin Monte Carlo (LMC), as recent work shows this to provide higher effective sample size per unit of computation time as compared to HMC [15].

The above process is repeated *i* = 1..*I* times to produce *I* independent samples from the posterior density. Because the samples are produced independently, without interaction among trajectories, the AIS algorithm is amenable to ‘embarrassing parallelization’ [20]. Specifically, trajectories can be assigned to individual computer processors or processor cores thus greatly speeding up the implementation.

Each sample is also accompanied by an importance weight

$$v^{\left(i\right)}=\frac{f_{1}\left(w_{1}\right)}{f_{0}\left(w_{1}\right)}\frac{f_{2}\left(w_{2}\right)}{f_{1}\left(w_{2}\right)}\frac{f_{3}\left(w_{3}\right)}{f_{2}\left(w_{3}\right)}\ldots \frac{f_{J}\left(w_{J}\right)}{f_{J-1}\left(w_{J}\right)}$$ (2)

which can be evaluated as

$$\log v^{\left(i\right)}=\sum_{j=1}^{J}\left(\beta_{j}-\beta_{j-1}\right)\log p\left(y|w_{j}\right)$$ (3)

To avoid numerical overflow we first create adjusted weights *u*_*i*_

$$\begin{array}{ccc} v_{max} & = & \max(\log v)\\ u_{i} & = & \exp(\log v^{\left(i\right)}-v_{max}) \end{array}$$ (4)

and let $\bar{u}$ be the mean adjusted weight. The normalised importance weights are

$$q_{i}=\frac{u_{i}}{\sum_{i}u_{i}}$$ (5)

A derivation of the formula for the importance weights is provided in [13] and included in S1 Text. The variance of the importance weights is an indicator of the quality of the approximation to the posterior density [13].

#### Annealing schedule

An important choice in any AIS implementation is the annealing schedule, that is, how to space the *β*_*j*_ over the (0, 1) interval. Calderhead and Girolami [21] show that, for estimates of the model evidence for linear regression models, the annealing schedule that minimises the Monte Carlo variance has a power-law form. Following [21, 22] the applications in this paper use a 5th-order geometric annealing schedule

$$\beta_{j}={\left(\frac{j}{J}\right)}^{5}$$ (6)

Additionally, one must choose the number of trajectories, and number of temperatures per trajectory. In the original AIS paper [13] *I* = 1000 trajectories were used with either *J* = 200 or 1000 temperatures. The AIS algorithm has also been compared to a Variational Bayes (VB) approach for scoring graphical models [23]. This implementation used only *I* = 5 trajectories with *J* = 16,384 temperatures. Proposals were made using a standard MH step which is perhaps one reason for the very large number of temperatures required. Only with *J* > 5000 temperatures did the AIS model evidence estimate exceed that produced by VB (which provides a provable lower bound [7]). In an application of AIS to score differential equation models [24], *I* = 10 trajectories with *J* = 40 temperatures were used along a 4th order geometric schedule, with a transition kernel implemented using an MH step with 4000 samples at each temperature. Because LMC provides better proposals than MH we envisage that a finer grained schedule can be used at similar computational expense. This will be examined in the results section in the context of linear and nonlinear regression models.

#### Model evidence

The importance weight, or the average importance weight across multiple trajectories, provides an approximation to the model evidence *p*(*y*|*m*) for model *m*, as shown below. This section uses the notation *p*(*y*|*w*, *m*) and *p*(*w*|*m*) to make it explicit that the likelihood and prior depend on model assumptions. We define the normalising constant at each temperature as

$$\begin{array}{ccc} Z_{j} & = & \int f_{j}\left(w\right)dw\\ & = & \int p{\left(y|w,m\right)}^{\beta_{j}}p\left(w|m\right)dw \end{array}$$ (7)

We then have

$$\begin{array}{ccc} Z_{0} & = & \int p(w|m)dw=1\\ Z_{J} & = & \int p(y|w,m)p(w|m)dw\\ & = & p(y|m) \end{array}$$ (8)

Therefore

$$\begin{array}{ccc} p(y) & = & \frac{Z_{J}}{Z_{0}}\\ & = & \frac{Z_{1}}{Z_{0}}\frac{Z_{2}}{Z_{1}}\frac{Z_{3}}{Z_{2}}\ldots \frac{Z_{J}}{Z_{J-1}}\\ & = & \prod_{j=0}^{J-1}r_{j} \end{array}$$ (9)

where *r*_*j*_ = *Z*_*j*+1_/*Z*_*j*_. We can then write

$$\begin{array}{ccc} r_{j} & = & \frac{1}{Z_{j}}\int f_{j+1}\left(w\right)dw\\ & = & \int \frac{f_{j+1}\left(w\right)}{f_{j}\left(w\right)}\frac{f_{j}\left(w\right)}{Z_{j}}dw\\ & \approx & \frac{1}{N}\sum_{n=1}^{N}\frac{f_{j+1}\left(w_{n}\right)}{f_{j}\left(w_{n}\right)} \end{array}$$ (10)

where the last line indicates a Monte-Carlo approximation of the integral with samples *w*_*n*_ drawn from the distribution at temperature *β*_*j*_. This can in turn be written as

$$r_{j}=\frac{1}{N}\sum_{n=1}^{N}p{\left(y|w_{n},m\right)}^{\beta_{j+1}-\beta_{j}}$$ (11)

For *N* = 1 we can therefore see that log *p*(*y*) is equal to Eq 3. To avoid numerical overflow we compute the log evidence as

$$\log p{\left(y|m\right)}_{AIS}=v_{max}+\log\bar{u}$$ (12)

We can now see that estimation of the model evidence using the Prior Arithmetic Mean (PAM) (see S1 Text), in which the average likelihood is computed over samples drawn from the prior, is a special case of the AIS estimate with just two temperatures, *β*_1_ = 1 and *β*_0_ = 0. It is also possible to define a reverse annealing schedule in which the temperature is gradually increased and defines a path from the posterior to the prior [13]. Agreement between forward and reverse estimates of the model evidence can then be used to ensure one has a sufficiently fine-grained annealing schedule [23]. For reverse schedules the Posterior Harmonic Mean (PHM) emerges as a special case of AIS with two temperatures (see S1 Text). AIS therefore generalises both PAM and PHM. In high dimensional spaces PAM underestimates the model evidence because it doesn’t sufficiently explore regions of high probability, whereas PHM overestimates it because it doesn’t sufficiently explore regions of low probability. These problems are ameliorated in AIS by the use of intermediate densities that form ‘bridges’ as described in a related method called bridge sampling [25].

In this paper our empirical results are based on forward annealing schedules only. Confidence intervals in model evidence estimates are provided using bootstrapping [26], by resampling the *I* estimates *N*_*boot*_ = 1000 times with replacement, computing the evidence for each, and finding the 5th and 95th percentiles. Thus, bootstrapping is implemented over trajectories.

### Langevin Monte Carlo

In this paper the transition densities *T*_*j*_ in AIS are implemented using a Langevin Monte Carlo (LMC) sampler, which leads to proposals being accepted with high probability even for nonlinear and high dimensional inference problems, as it uses information about the gradient and curvature of the unnormalised density, *f*_*j*_.

The use of LMC follows from the definition of the log joint and its gradient as a function of *w*

$$\begin{array}{ccc} L(w) & = & \log p(y|w)+\log p(w|\mu ,\Lambda )\\ g(w) & = & \frac{dL(w)}{dw} \end{array}$$ (13)

A proposal is drawn as

$$\begin{array}{ccc} w_{s}^{*} & ∼ & p(w_{s}^{*}|w_{s})\\ p(w_{s}^{*}|w_{s}) & = & N(w_{s}^{*};m,C)\\ m & = & w_{s}+\frac{1}{2}Cg\left(w_{s}\right)\\ C & = & h^{2}{\left.(\Lambda +F\right)}^{-1} \end{array}$$ (14)

where Λ is the prior precision, *w*_*s*_ is the *s*th sample, and *h* is a step size parameter (fixed at 0.5 for all applications in this paper). The quantity *F* is the Fisher Information matrix (see S2 Text) and quantifies the precision of the parameters conferred by the data. This has analytic forms for many probabilistic models such as logistic regression [14] and is readily computed for differential equation models using an approach based on forward sensitivity analysis [27, 28].

The Metropolis-Hastings (MH) criterion is then applied to accept proposals with probability

$$r=\frac{p_{w}\left(w_{s}^{*}\right)}{p_{w}\left(w_{s}\right)}\frac{p(w_{s}|w_{s}^{*})}{p(w_{s}^{*}|w_{s})}$$ (15)

where *p*_*w*_(*w*_*s*_) = exp[*L*(*w*_*s*_)]. The proposal is always accepted if *r* > 1. We set $w_{s+1}=w_{s}^{*}$ if the sample is accepted and *w*_*s*+1_ = *w*_*s*_ if it is rejected.

The above proposal (Eq 14) has the same functional form as the Simplified Manifold MALA algorithm as applied to ODEs [14, 27]. Here the ‘manifold’ is defined by *C* and *m* and its computation has been ‘simplified’ as the curvature has been assumed to be locally constant. For Gaussian likelihoods, this same local linearity assumption is also the basis of the Gauss-Newton optimization algorithm [29].

In the usual application of LMC [14, 15], Eqs 14 and 15, are repeatedly applied until one obtains samples from the posterior density. However, in this paper we use LMC to provide a single sample at each temperature in an AIS trajectory. Specifically, the transition kernel, *T*_*j*−1_(*w*_*j*_|*w*_*j*−1_), starts at *w*_*s*_ = *w*_*j*−1_ and produces $w_{j}=w_{s}^{*}$ using the modifield log joint *L*_*j*−1_. This modification requires multiplication of the likelihood, gradient and Fisher information by *β*_*j*−1_. The LMC updates are otherwise identical. Because LMC is used to produce only a single sample at each temperature the total number of LMC steps is equal to the number of temperatures.

We now briefly comment on the computational scalability of the combined AIS-LMC algorithm. Because AIS is based on importance sampling its accuracy is proportional to the number of annealing runs (“trajectories”) [13]. As trajectories are independent, and can be assigned to cores on multiple core computer architectures, the accuracy will therefore scale with the number of cores (at almost no increase in computer time). For a fixed number of cores computer time scales linearly with the number of trajectories. The computational bottleneck within each AIS trajectory is the evaluation of the gradient of the log joint and the Fisher information, required for each LMC step. These quantities can be efficiently computed for ODE models using forward sensitivity or adjoint methods [27, 28]. The computation time of these methods scales linearly with the length of time series being modelled, and adjoint methods are typically more efficient than forward sensitivity methods if the number of parameters is much larger than the number of dynamical states.

### Linear Regression

In multiple linear regression an [*N* × 1] data vector *y* is generated as

y=Xβ+e (16)

where *X* is an [*N* × *p*] design matrix, *β* is a [*p* × 1] vector of regression coefficients, and *e* is an [*N* × 1] zero-mean IID Gaussian noise vector with entries having variance *σ*^2^.

### Nonlinear Regression

To provide a simple nonlinear model with multiple maxima, we consider a regression model where the parameters of interest are nonlinearly related to the regression coefficients

$$\begin{array}{ccc} y & = & \sum_{i}x_{i}\beta_{i}+e\\ \beta_{i} & = & w_{i}^{2} \end{array}$$ (17)

This model will have multiple maxima over the various combinations of positive and negative values of *w*_*i*_.

We also consider an exponential approach-to-limit or ‘approach’ model where

$$y\left(t\right)=-60+V_{a}\left[1-\exp(-t/\tau )\right]+e\left(t\right)$$ (18)

with parameters *w*_1_ = log *τ* and *w*_2_ = log *V*_*a*_. This models the ramping up of a voltage from −60 to −60 + *V*_*a*_ with a time constant *τ*, and has the same mathematical form as Biochemical Oxygen Demand (BOD) models [30] previously used to evaluate Bayesian inference methods [31].

### Neural Mass Models

#### Single region

In Neural Mass Models (NMMs) [17], postsynaptic potentials (PSPs) at excitatory synapses are related to firing rates via convolutions with synaptic kernels

$$v_{out}\left(t\right)=h_{e}\left(t\right)⊗s\left(v_{in}\right)$$ (19)

where the population firing rate function

$$s\left(x\right)=\frac{1}{1+\exp(-r_{1}\left(x-r_{2}\right))}-\frac{1}{1+\exp(r_{1}r_{2})}$$ (20)

has parameters *r*_1_ and *r*_2_, and the synaptic kernel is given by an alpha function

$$h_{e}\left(t\right)=\frac{H_{e}}{\tau_{e}}t\exp\left(-t/\tau_{e}\right)$$ (21)

with magnitude *H*_*e*_ and time constant *τ*_*e*_. Inhibitory synapses are similarly defined but with kernels *h*_*i*_(*t*) and parameters *H*_*i*_, *τ*_*i*_.

The activity of a single neocortical unit is then defined by the convolution equations

$$\begin{array}{ccc} v_{i} & = & \gamma_{3}s\left({\tilde{v}}_{p}\right)⊗h_{e}\\ v_{s} & = & \left[s\left(u\right)+\gamma_{1}s\left({\tilde{v}}_{p}\right)\right]⊗h_{e}\\ v_{pe} & = & \gamma_{2}s\left({\tilde{v}}_{s}\right)⊗h_{e}\\ v_{pi} & = & \gamma_{4}s\left({\tilde{v}}_{i}\right)⊗h_{i}\\ v_{p} & = & v_{pe}-v_{pi} \end{array}$$ (22)

where *v*_*pe*_ and *v*_*pi*_ are potentials at excitatory and inhibitory synapses in the pyramidal cell population, $\tilde{v}$ denotes the potential after a delay *δ*_*ii*_ due to signalling delays among the different populations within a single brain region. Following [17] a first order Taylor series approximation is used to capture these delays, $\tilde{v}=v-\delta_{\mathrm{ii}}\dot{v}$. The connection strengths among neural populations are specified by the parameters *γ*_1..4_. These within-region values are also referred to as the ‘intrinsic connectivity’.

Each of the above convolution equations can be written as a second order differential equation, or two first order DEs, as shown in [12] (see also S3 Text). Thus a single cortical unit has *N*_*x*_ = 9 state variables. The input to the cortical region, *u*, is a surrogate for event-related subcortical brain activity and is specified by a Gaussian function peaking at 64ms post-stimulus with width 16ms.

#### Two region model

**Fig 1:**
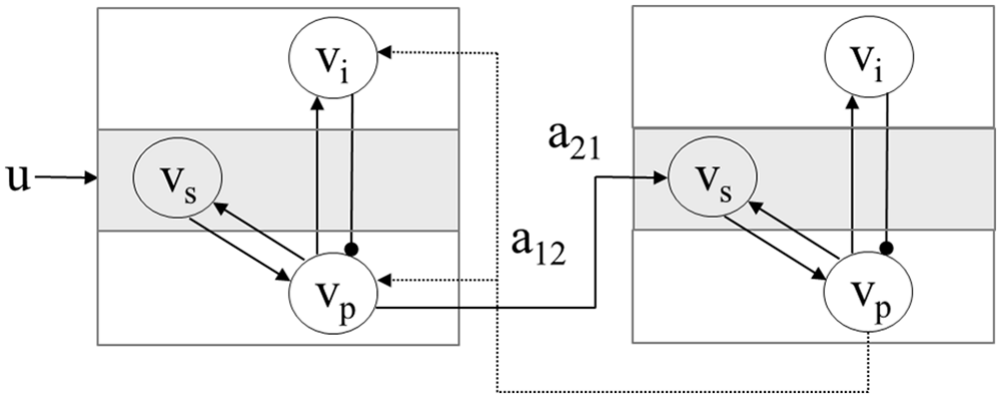
Neural mass model of two cortical regions in a hierarchical network. The first unit receives thalamic input *u*, and projects output *v*_*p*_(1) via a forward connection of strength *a*_21_ to region 2. The second unit produces output *v*_*p*_(2) and projects it via a backward connection of strength *a*_12_ to region 1.

David et al. [17] describe how cortical units can be connected into hierarchical networks that follow known anatomical connectivity patterns [32]. A two region network with forward connection *a*_21_ (from region 1 to 2) and backward connection *a*_12_ is shown in Fig 1. The convolution equations for this network are given in S3 Text.

There are two between-region or ‘extrinsic’ connectivity parameters (*a*_12_, *a*_21_) and two extrinsic delay parameters (*δ*_12_ and *δ*_21_). Additionally, we have four ‘intrinsic’ connectivity parameters (*γ*_1..4_) and parameters of firing rate functions (*r*_1_ and *r*_2_) that are constrained to be identical in each region. This gives a total of *N*_*p*_ = 10 neurophysiological variables to estimate.

The intrinsic delay parameters (*δ*_11_, *δ*_22_—one for each region) are assumed known. The synaptic time constants (*τ*_*e*_, *τ*_*i*_) and synaptic response magnitudes (*H*_*e*_, *H*_*i*_) are fixed to be the same for all regions, and are also assumed known. This two region neural mass model has *N*_*x*_ = 18 state variables. The differential equations are integrated to produce time series of currents and potentials for each population in each cortical unit, at *N*_*t*_ time points. The resulting ‘neuronal state matrix’ *X* is of dimension [*N*_*x*_ × *N*_*t*_]. The generative model is then specified as

$$Y=L_{2}X+e$$ (23)

where *L*_2_ is a matrix that picks off the pyramidal cell activities in each of the regions, and *e* is zero mean Gaussian noise. For the simulations in this paper *Y* is therefore a [2 × *N*_*t*_] data matrix containing the pyramidal cell activities of each of the brain regions. In applications to empirical M/EEG data [17, 33] an [*N*_*d*_ × *N*_*x*_] lead field matrix *L* is used to model Event-Related Potentials (ERPs) at *N*_*d*_ sensors.

We assume that the noise variance on the *s*th output (where *s* = 1..2) is $\sigma_{s}^{2}$. The model likelihood is therefore

$$p\left(Y|w\right)=\prod_{t=1}^{T}N\left(y_{t};{\hat{y}}_{t},C_{e}^{-1}\right)$$ (24)

where *w* are the parameters, ${\hat{y}}_{t}=L_{2}x_{t}$ and $C_{e}=\text{diag}\left(\sigma_{s}^{2}\right)$. The unknown neurophysiological variables are related to model parameters according to the transformations shown in S1 Table which enforce positivity and constrain parameters within a physiologically plausible range. The Gaussian prior over model parameters has zero mean *μ*, and Λ^−1^ is a diagonal matrix with entries of 0.16 for the first two parameters (*a*_12_ and *a*_21_) and 0.0625 for the rest. The above choice of parameter transformation and prior are the same as that used in DCM for ERP [33].

### Testing for Normality

As the VL algorithm assumes that the posterior distribution is Gaussian it will be interesting to see if this is indeed the case. We use Royston’s test for multivariate normality [34] using a Matlab implementation by Trujillo-Ortiz et al [35]. This is a multivariate extension of the Shapiro-Wilks test and we apply it to Monte Carlo samples from the posterior densities produced by AIS. As these samples are independent there is no need for ‘thinning’ or assessments of Effective Sample Size [36].

### Software

The algorithms on which this research is based have been implemented in Matlab in the ‘Monte Carlo Inference (MCI)’ toolbox and will be distributed as part of a forthcoming release of the Statistical Parametric Mapping (SPM) package. AIS and LMC, for example, are implemented in the spm_mci_ais.m and spm_mci_lgv.m functions available in the subdirectory /toolbox/mci/inference/.

### Variational Laplace

The Variational Laplace (VL) algorithm is instantiated in the SPM software [33] (in the function spm_nlsi_GN.m) and described elsewhere [6, 37]. We also include a brief mathematical description in S4 Text. In VL, the posterior is assumed to factorise into a product of probability distributions, one over latent variables controlling noise variances and one over model parameters. Each distribution is multivariate Gaussian with mean and covariance that are iteratively updated to maximise an approximation to the model evidence [6]. Importantly, the multivariate nature of each Gaussian allows parameter dependencies to be accommodated. This optimiser is the standard approach used for the majority of DCM applications in neuroimaging. Known noise variances (see below) are implemented for the VL algorithm by setting the prior over the log noise precision to have a mean corresponding to the true (known) value, and a variance of 10^−8^ (i.e. very tight).

By default, the implementation of VL in SPM initialises parameters at the prior mean. A simple way of potentially handling optimisation problems with multiple maxima, however, is to run the VL algorithm multiple times where each run is initialised using a different sample from the prior. We will refer to this procedure as Multistart VL.

## Results

We present results on linear and nonlinear regression models to demonstrate the effect of the number of temperatures *J* and trajectories *I* in AIS. The algorithms were run on a high-end desktop computer (Hewlett Packard Z440) with 32G memory, 8 cores, and a 64-bit operating system. All the results are derived from synthetic data for which the ground truth parameters are known. Following other recent comparisons of inference algorithms for differential equation models [15, 21, 38, 39], our simulations assume that the noise variances are known for models with Gaussian likelihoods.

All AIS results were produced using a fifth order geometric annealing schedule and the posterior mean was computed using the mean over trajectories. The AIS implementation was parallelized using the Matlab Parallel Computing toolbox such that independent ‘pool workers’ (in this case cores) were assigned to different trajectories. The distribution of normalised importance weights, *u*_*i*_, is characterised in two ways. Firstly, by the entropy. For *I* trajectories the maximum entropy is log_2_ *I* e.g. 5 bits for *I* = 32. Secondly, by the number of significantly non-zero values, *I*_*q*_, which we define as the number above 0.01.

### Linear Regression

We first provide results on a multiple linear regression model, as there are analytic formulae for the posterior distribution and model evidence [7], and the Laplace approximation is exact. This comprised *p* = 7 regressors chosen from a discrete cosine basis set over *N* = 20 ‘time points’, with additive noise of standard deviation *σ* = 0.2. The prior variances, $\Lambda_{pp}^{-1}$ were set to 10 for each regressor and the prior means, *μ*_*p*_ to zero. The regression coefficients were drawn from the prior.

The AIS algorithm was applied to this data using *J* = 512 temperatures and *I* = 32 independent samples. We fitted the true model (with 7 regressors) and a reduced model to the same data but this time using only the first 6 regressors.

Using the 32 samples produced by AIS, we could not reject the hypothesis that the posterior was Gaussian using Royston’s test for the full (*p* = 0.67) and reduced (*p* = 0.68) models. This is of course to be expected as the posterior distribution is indeed Gaussian for linear regression models [7]. For the full model, the normalised importance weights had high entropy, *H* = 4.07, and many trajectories had significant weight, *I*_*q*_ = 21.

**Fig 2:**
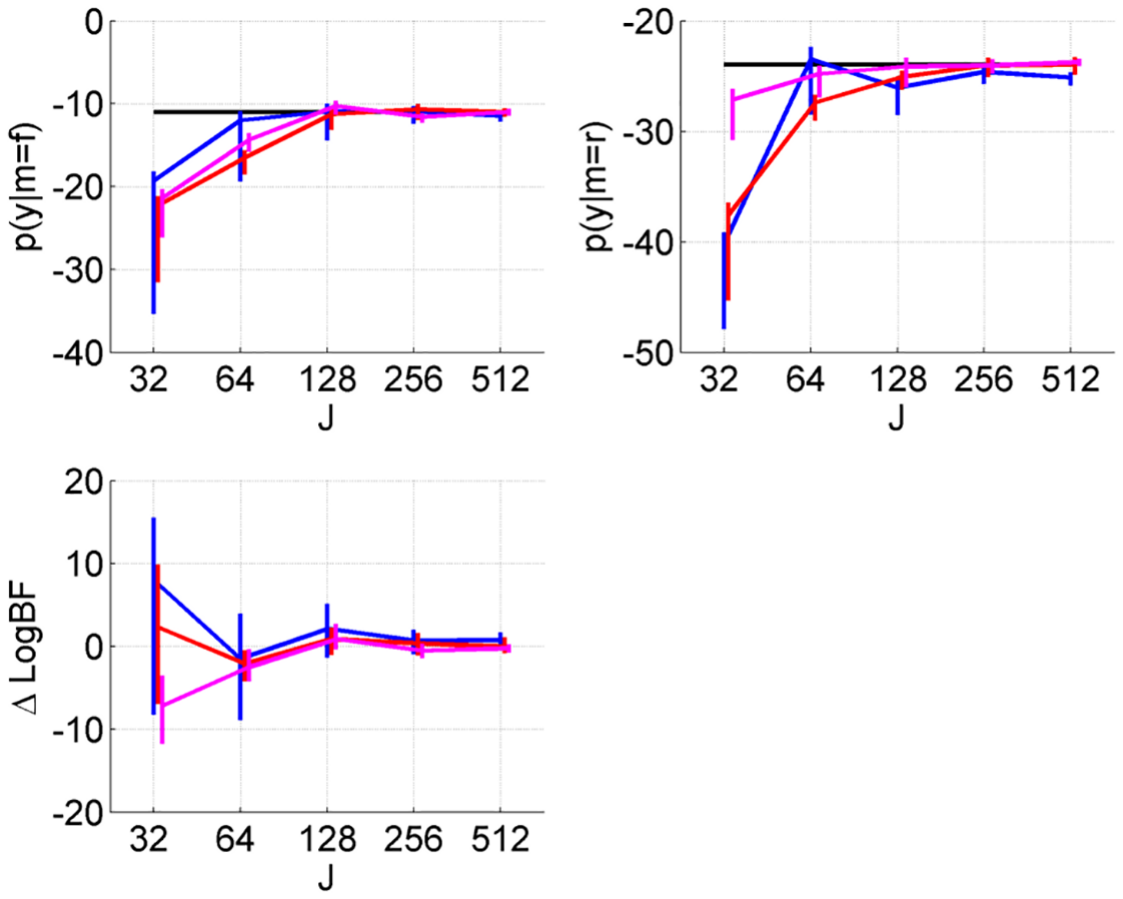
Linear regression. AIS approximations of log evidence for a ‘full’ model with 7 parameters (top left), and a ‘reduced’ model with 6 parameters (top right) as a function of number of temperatures *J*. These approximations use *I* = 16 (blue), *I* = 32 (red) and *I* = 64 (magenta) trajectories. The black lines show the equivalent analytic quantities. The bottom left plot shows the difference between the AIS estimated log Bayes factor and the true value. Vertical lines span the 5th and 95th percentiles from bootstrapping.

The AIS estimates of the log model evidences for the full, log *p*(*y*|*m* = *f*), and reduced models, log *p*(*y*|*m* = *r*) and the corresponding log Bayes factor, and computation times, are provided in Table 1. The estimates very closely match the analytic values. Note that the VL estimates correspond to the analytic values for the case of linear regression [7]. We then re-estimated the evidences using different numbers of AIS samples and temperatures, with results plotted in Fig 2. Theses results show good agreement with analytic values for *J* = 128 and above. The error bars on AIS model evidence estimates were computed using bootstrapping (over trajectories) as described in the section on ‘Annealed Importance Sampling’, in the subsection on ‘Model Evidence’.

**Table 1:** Evidence and Bayes Factor Approximations (Single Run).

Model	Estimate		Time(s)	
	VL	AIS	VL	AIS
Linear, LogEv, Full	-11.02*	-11.00	0.005	15.4
Linear, LogEv, Red	-23.97*	-23.94	0.002	3.1
Linear, LogBF	12.95*	12.94	-	-
Approach, LogEv, Full	-73.88	-73.77	0.58	19.4
Approach, LogEv, Red	-783.62	-783.61	0.02	2.9
Approach, LogBF	709.74	709.84	-	-
Neural Mass, LogEv, Full	1524.1	1563.6	22	5290
Neural Mass, LogEv, Red	1288.4	1293.4	24	4610
Neural Mass, LogBF	235.74	270.2	-	-

These results are for a single run of each inference algorithm (AIS or VL). AIS estimates from *I* = 32 samples and *J* = 512 trajectories. The results for the linear model here* are for the analytic solution, which also corresponds to the VL solution.

### Nonlinear Regression

#### Multiple maxima

**Fig 3:**
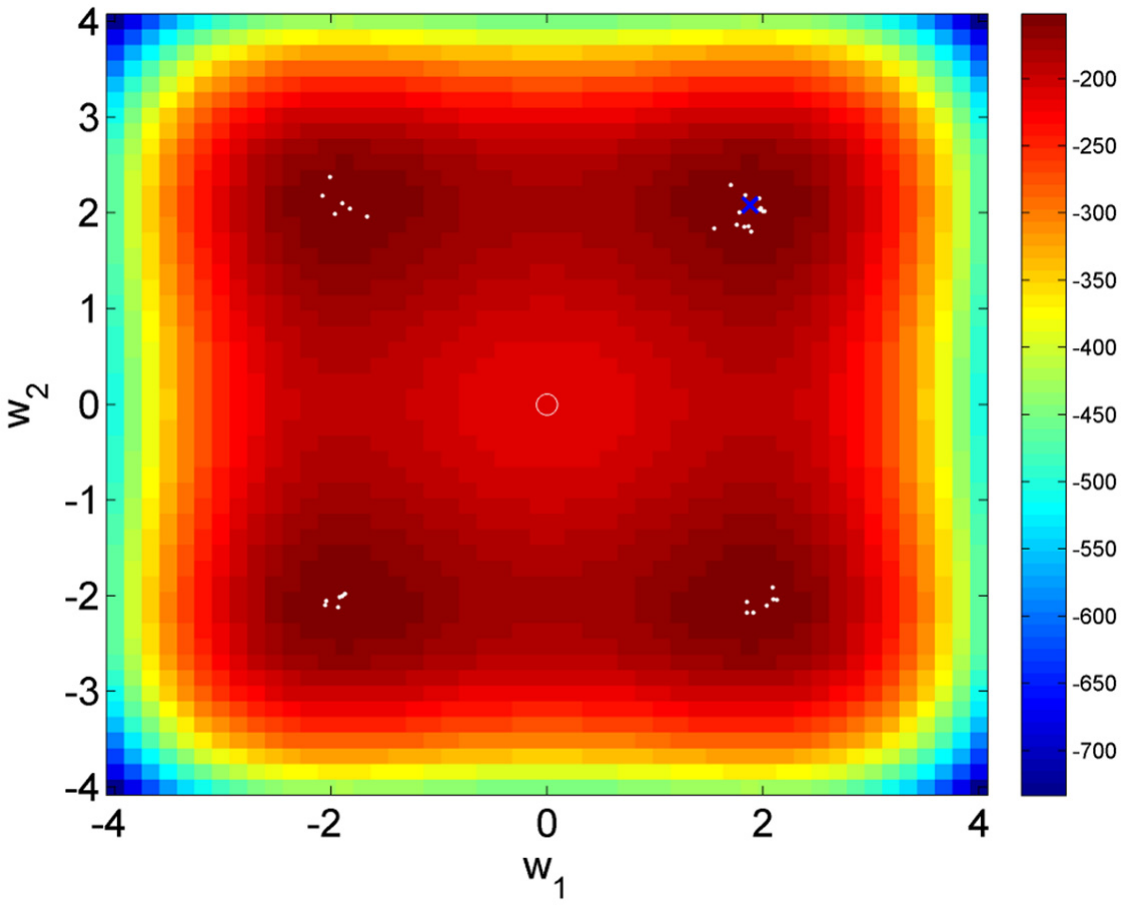
Log posterior of nonlinear regression model with multiple maxima. The true parameters are *w*_1_ = *w*_2_ = 2. The circle denotes the prior mean. Samples from the posterior density as computed using AIS are shown as white dots (in each of the four maxima), and the blue cross close to the true parameters denotes the VL posterior mean.

We now report results for the nonlinear regression model that was designed to have multiple maxima. This model has two independent variables, *x*_1_ and *x*_2_, corresponding to two components of a discrete cosine basis set (the first two from the linear regression problem described above). The priors were set to be the same as for the linear regression simulation but the observation noise was increased to *σ* = 0.5. AIS was run using the same parameters as before and Fig 3 shows samples from the posterior density which lie in all of the four posterior modes. The normalised importance weights had lower entropy than for the linear regression model, *H* = 3.42, and fewer trajectories with significant weight, *I*_*q*_ = 16. We can reject the hypothesis that the posterior is Gaussian using Royston’s test (*p* = 10^−12^). Thus AIS is able to accomodate multiple maxima as expected, and we correctly infer that the posterior is non-Gaussian. AIS is able to find the different maxima by virtue of employing multiple trajectories. Fig 3 also shows the posterior mean for VL. We also ran Multistart VL (see section on Variational Laplace) with 32 starts and, as expected, it was also able to identify each of the four maxima. The posterior distribution for this example is multimodal and is therefore not well represented by the posterior mean. The AIS samples do, however, collectively provide a good description of the posterior distribution.

#### Approach to limit

We now report results for the approach-to-limit model. Data were generated with parameters *V*_*a*_ = 30, *τ* = 8 and Gaussian observation noise variance of unity. The prior has mean *μ* = [3, 1.6]^*T*^ and precision Λ = diag ([16, 16]). A ‘reduced’ model was defined as only having the *V*_*a*_ parameter, thus producing a constant prediction over the time interval.

The AIS algorithm was applied to this data using *J* = 512 temperatures and *I* = 32 independent samples. Using the 32 samples produced by AIS, we could not reject the hypothesis that the posterior was Gaussian using Royston’s test (*p* = 0.96). The estimates of the model evidences and Bayes factors, shown in Table 1, agree very well with those from VL. The normalised importance weights had high entropy, *H* = 4.27, and many trajectories had significant weight, *I*_*q*_ = 21.

**Fig 4:**
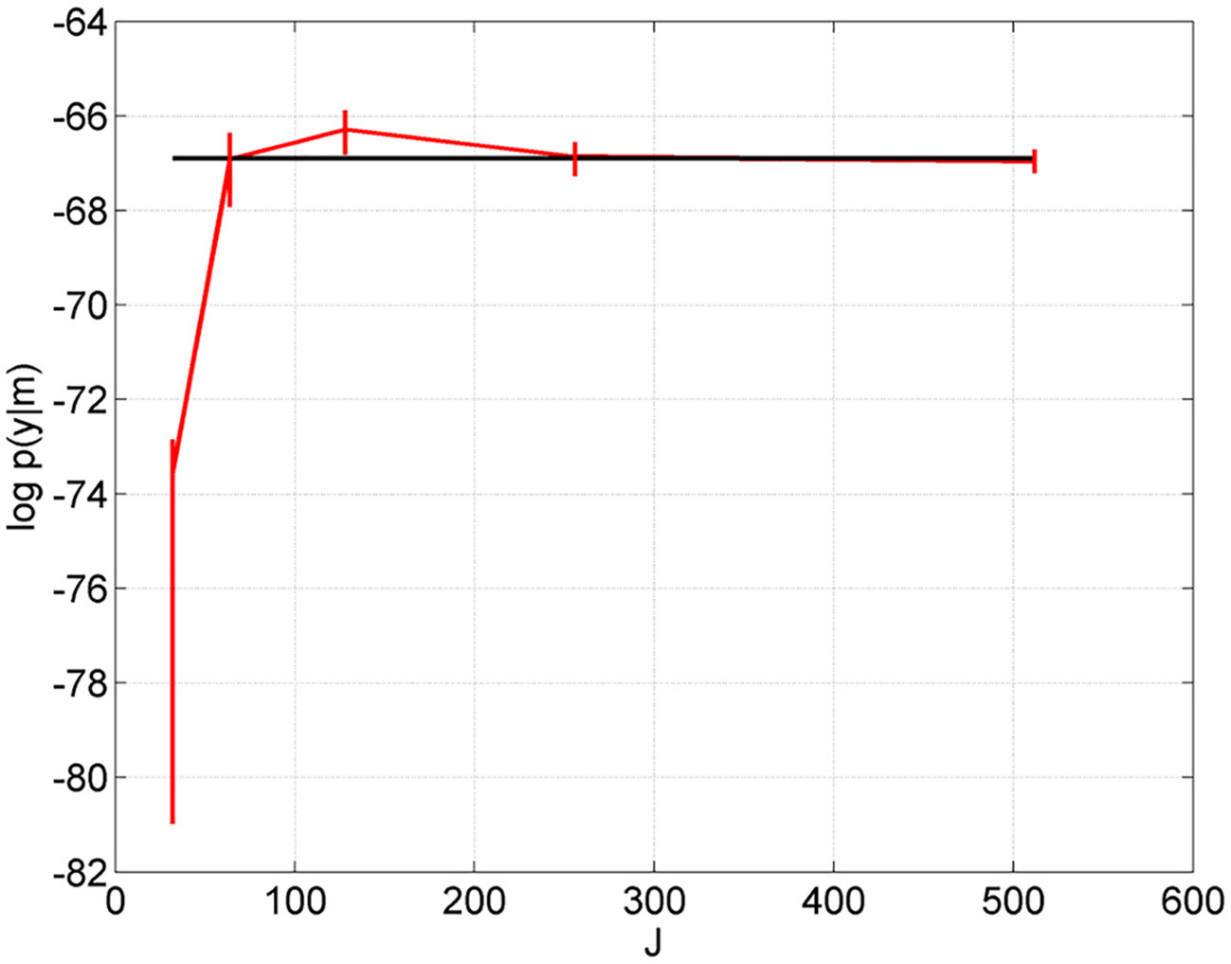
Approach-to-limit model. AIS approximation of log evidence (red line) as a function of number of temperatures *J*. Vertical lines span the 5th and 95th percentiles from bootstrapping. These approximations use *I* = 32 samples. The VL approximation is shown as the black line.

Fig 4 shows the AIS approximation to the log model evidence, using *I* = 32 samples, as a function of the number of temperatures *J*. We see good agreement with VL for *J* larger than 128. These simulation results were based on a second data set from the approach model created by sampling parameters from the prior and producing time series as above (because this is a different data set the log evidence values are different to those in Table 1).

### Neural Mass Models

To produce the following results the differential equations underlying the neural mass models (see S3 Text) were integrated using implicit backward-differentiation formulas (BDFs) and the resulting nonlinear equations solved using Newton’s method as implemented in the CVODES software [40]. With a relative tolerance of 10^−2^ and an absolute tolerance 10^−4^ this algorithm took an average of 75ms (averaged over ten runs) to produce the time series for the two-region model. This was lower than the 229ms for Matlab’s ODE15s integrator and the 90ms for SPM’s (implemented in the function spm_int_L.m). Both VL and AIS model estimation approaches therefore used the CVODES implementation. For the LMC algorithm used in AIS, gradients were computed using a forward sensitivity method as implemented in CVODES. For VL, gradients and curvatures were computed using central differences as implemented in the SPM function spm_nlsi_GN.m.

**Fig 5:**
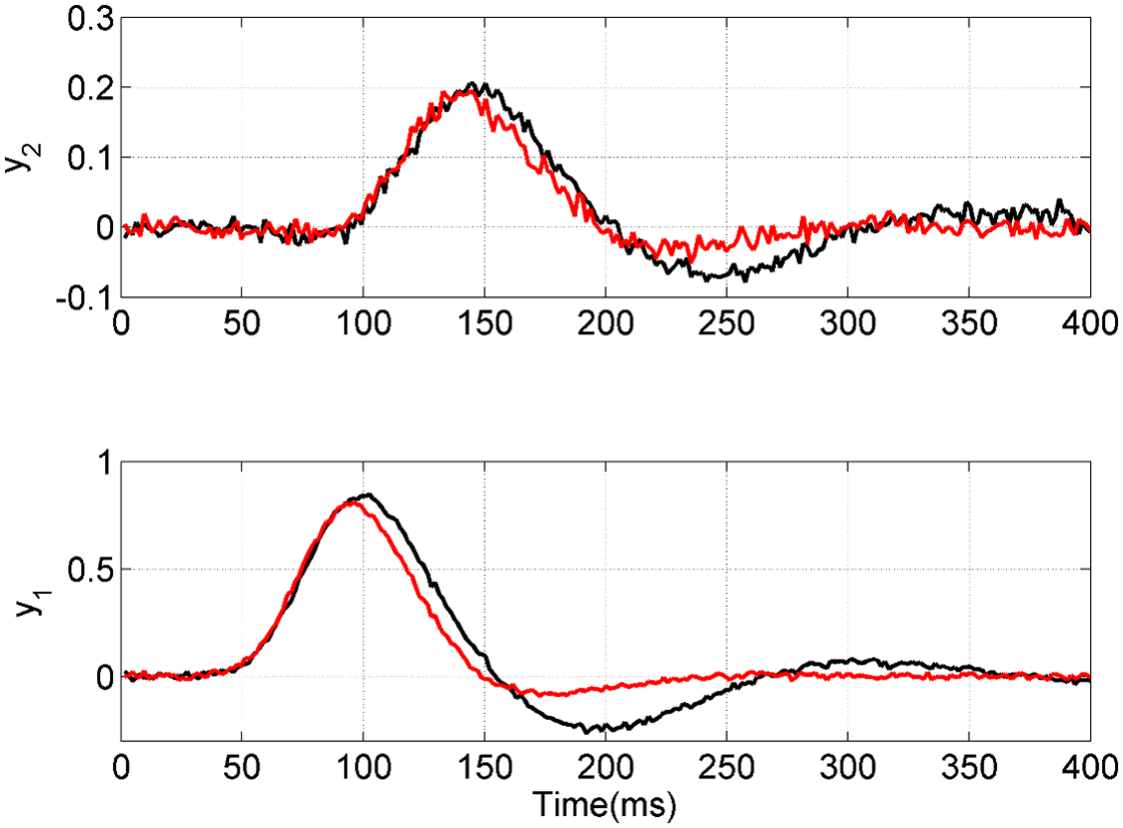
Time series from neural mass models. The bottom figure shows the pyramidal cell potential in region 1 for the full model (black) and reduced model (red). The top figure shows the same for the pyramidal cells in region 2. The reduced model is identical to the full model except that it does not have the backward connection from region 2 to 1. All time series contain additive Gaussian observation noise with standard deviation *σ*_*s*_ = 0.01.

The simulations that follow make use of the two-region neural mass model depicted in Fig 1 and described above. We generated data from a model with strong forward and backward connections. This is specified using the parameter values *w*_1_ = *w*_2_ = 1 which set the connections *a*_21_ and *a*_12_ according to S1 Table. The other parameters were set to zero. Data was then generated from the model as described above using zero mean additive Gaussian noise having standard deviation *σ*_*s*_ = 0.01. The resulting time series are shown in black in Fig 5. The priors over model parameters for Bayesian model fitting are as described at the end of the above subsection ‘Two-Region Model’ in the section on ‘Neural Mass Models’.

We then fitted two models to the data using AIS, a ‘full’ model, which has the same structure as the model from which the data were generated, and a ‘reduced’ model which did not have the backward connection. We used *I* = 32, *J* = 512 and model estimation took 5290s and 4610s for the full and reduced models. The estimated log model evidences were 1563.6 for the full model and 1293.4 for the reduced model, corresponding to a Log Bayes Factor of 270.2 in favour of the full model. Using the 32 samples produced by AIS, we could not reject the hypothesis that the posterior was Gaussian using Royston’s test for the full (*p* = 0.32) and reduced (*p* = 0.15) models.

The AIS acceptance rates, *a*_*j*_, averaged over the *I* = 32 trajectories, showed a gradual decrease with *β*_*j*_. Averaging *a*_*j*_ over the high temperatures (*β*_*j*_ < 0.5) gave a value of *a*_*high*_ = 0.43 and over the low temperatures of *a*_*low*_ = 0.19. These acceptance rates show that the cost function is being sufficiently explored and are in line with other Bayesian annealing methods [38]. The normalised importance weights had lower entropy than for the previous models above, *H* = 2.59, and fewer trajectories with significant weight, *I*_*q*_ = 12.

We also fitted the full and reduced models using VL, which took 22s and 24s (using 19 and 22 VL iterations) respectively. The estimated log model evidences were 1524.1 for the full model and 1288.4 for the reduced model, corresponding to a Log Bayes Factor of 235.74 in favour of the full model. Thus, the VL and AIS estimates agree reasonably well for the reduced model (within 0.4 per cent) but not for the full model (within only 2.5 per cent). Which are we to believe?

As described in S1 Text, it is also possible to use the VL posterior as a proposal density to provide an importance sampling estimate of the model evidence, without using any annealing. We refer to this procedure as ISVL and used it to generate 1000 samples. ISVL is highly computationally efficient, requiring only 90s of compute time. The estimate of the log evidence was 1562.8 for the full model which agrees very well with the AIS estimate (within 0.05 per cent).

**Fig 6:**
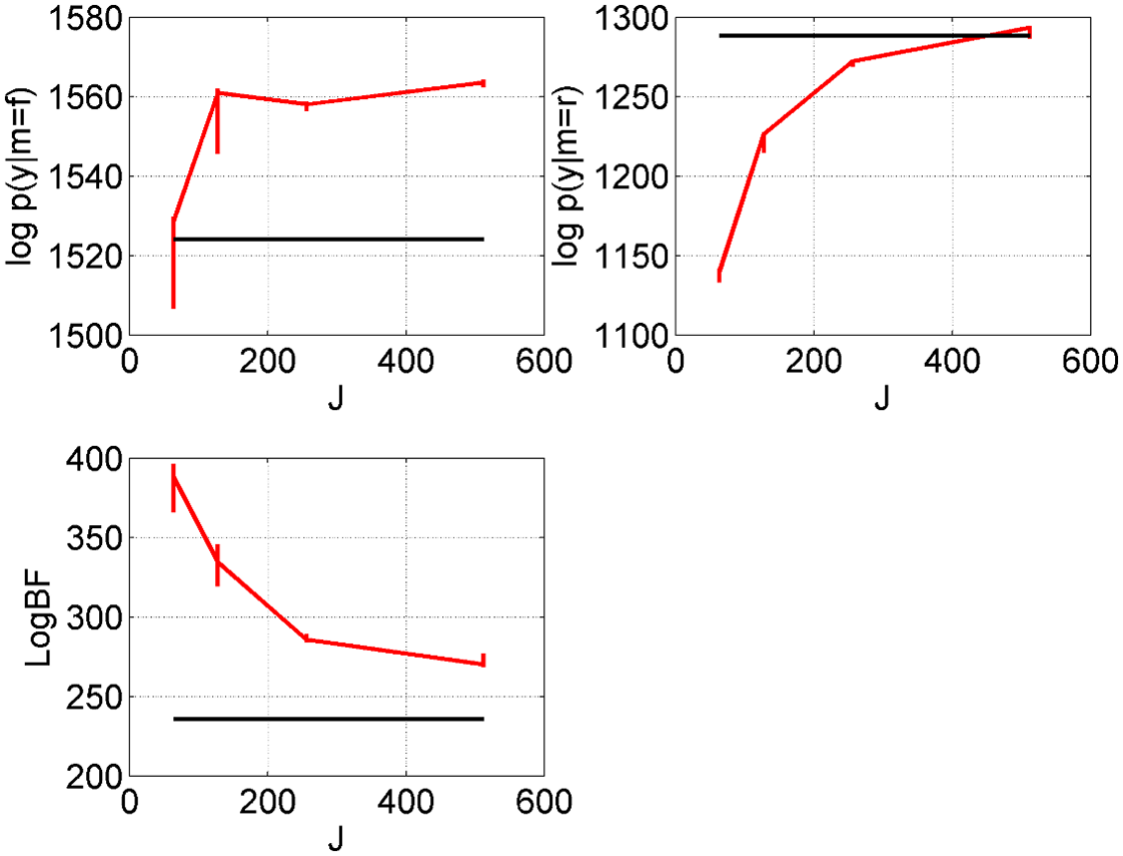
Two-region neural mass model: Temperature discretisation. The red lines indicate the AIS approximation of log evidence for full model, log *p*(*y*|*m* = *f*) (top left), log evidence for reduced model, log *p*(*y*|*m* = *r*) (top right), and log Bayes factor for full versus reduced, as a function of number of temperatures *J*. The vertical lines span the 5th to 95th percentiles from bootstrapping. These approximations use *I* = 32 samples. The black lines show the equivalent quantities for VL.

Fig 6 plots the log evidences and log Bayes factor as a function of the number of temperatures *J*. These indicate that a fine-grained temperature resolution *J* is required to obtain good results. We also note that the log joint probability, *L* (see Eq 13), of the posterior mean AIS solution increases with *J*, with values of *L* = 1583, 1584, 1588, 1589 for *J* = 64, 128, 256, 512. The log joint probability of the true parameters is *L* = 1589, whereas the log joint of the VL posterior mean is only *L* = 1157.

**Fig 7:**
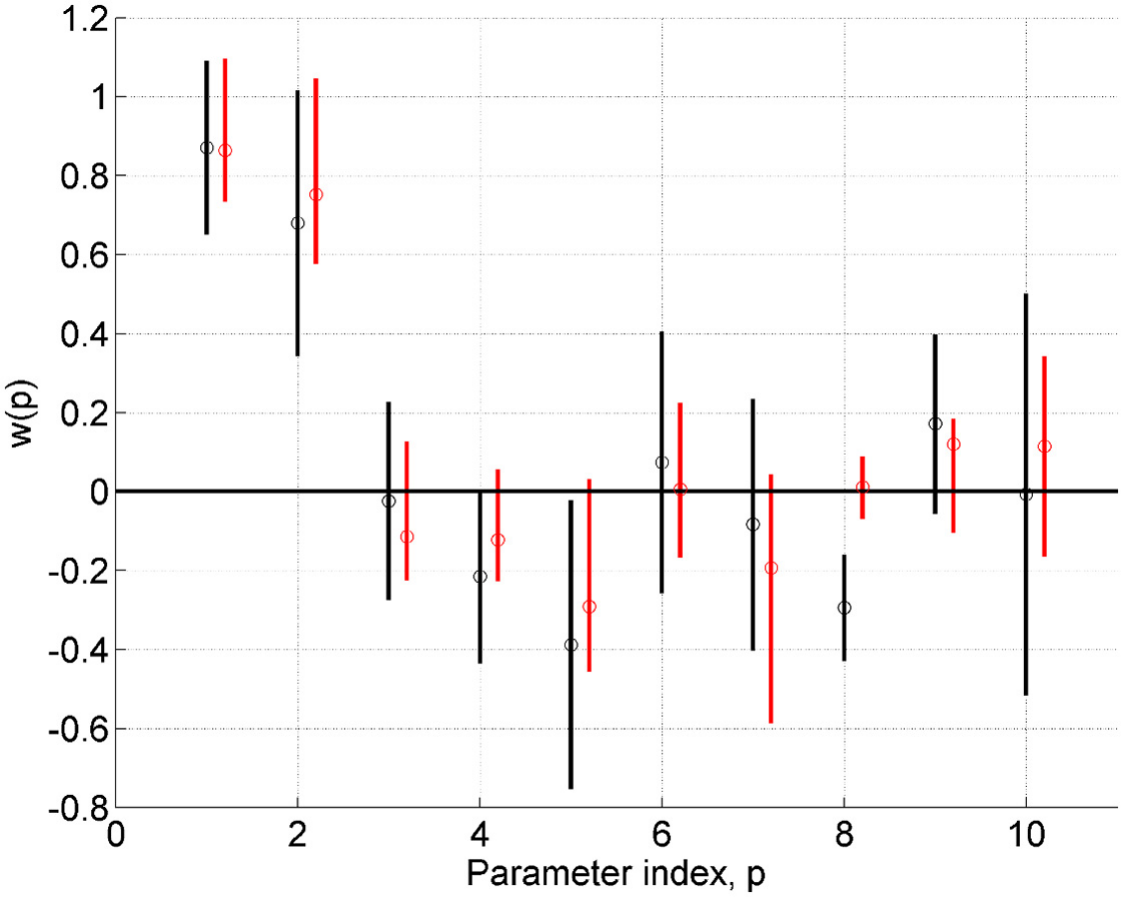
Two-region neural mass model: Posterior densities. Univariate posterior densities from a single model fit for AIS (red) and VL (black). The vertical lines span the 5th to 95th percentiles and the circles denote the posterior means. AIS provides better estimates for eight out of ten parameters.

Fig 7 plots the posterior densities from fitting the full model for VL and the AIS solution with *J* = 512. The estimates are generally in agreement but the AIS posterior means are closer to the true parameter values (*w*_1_ = *w*_2_ = 1, *w*_3_ to *w*_10_ equal to 0) for eight out of ten parameters. This is reflected in the higher joint probability mentioned above. Given that we know the true parameters we can also compute the Root Mean Squared Error (RMSE) between true and posterior mean parameters. For VL this is 0.21 and for AIS it is 0.11.

#### Multiple runs

Perhaps it is not surprising that AIS has found a better solution given that it requires 240 times as much computer time (for the full model and with *J* = 512). We therefore compared AIS to a Multistart VL procedure (see above description in the section ‘Variational Laplace’) using 240 multi-starts, so as to equate computation time with AIS. The best solution had a log joint of *L* = 1428. The remaining solutions had a log joint of less than *L* = 1275, with 84% having 1130 ≤ *L* ≤ 1175. Our initial solution (with *L* = 1157) is therefore fairly typical. On this evidence multi-start VL doesn’t seem to be the best strategy.

Both AIS and VL will produce slightly different results over different runs of the algorithm (sampling trajectories for AIS, initialisations for VL). To quantify this variation we ran each algorithm twenty times and report the mean results and standard deviations for estimates of the log Bayes Factors, log joint density and RMSE in tables 2, 3 and 4.

**Table 2:** Evidence and Bayes Factor Approximations (Multiple Runs).

Model	VL	AIS	AMC4-PHM	AMC4-Chib
Linear, LogEv, Full	-11.02*	-11.07 (0.39)	-0.62 (3.49)	-11.31 (0.48)
Linear, LogEv, Red	-23.97*	-24.00 (0.31)	-14.09 (1.84)	-24.06 (0.24)
Linear, LogBF	12.95*	12.94 (0.49)	13.48 (3.49)	12.75 (0.56)
App, LogEv, Full	-60.85 (0.02)	-60.85 (0.27)	-57.42 (0.29)	-60.86 (0.04)
App, LogEv, Red	-662.67 (0.00)	-666.52 (0.13)	-664.36 (0.59)	-666.53 (0.02)
App, LogBF	605.68 (0.02)	605.67 (0.33)	606.94 (0.63)	605.67 (0.05)
NMM, LogEv, Full	1524.11 (0.00)	1563.12 (1.22)	1405.94 (130.88)	1476.05 (50.73)
NMM, LogEv, Red	1288.37 (0.00)	1291.26 (5.10)	63.81 (567.51)	451.93 (541.37)
NMM, LogBF	235.7 (0.01)	271.86 (5.27)	1342.13 (599.68)	1024.13 (559.24)

AIS estimates from *I* = 32 samples and *J* = 512 trajectories. The results for the linear model here* are for the analytic solution, which also corresponds to the VL solution. Entries shows the mean values from 20 runs of each algorithm with standard deviations shown in brackets. ‘App’ denotes the Approach model and ‘NMM’ the two-region neural mass model.

**Table 3:** Log Joint Density of Posterior Mean (Multiple Runs).

Model	VL	AIS	AMC4
Linear, Full	-11.74*	-11.87 (0.06)	-12.07 (0.40)
Linear, Red	-24.67*	-24.78 (0.06)	-24.78 (0.10)
App, Full	-54.83 (0.02)	-54.97 (0.03)	-54.83 (0.00)
App, Red	-662.67 (0.00)	-662.70 (0.05)	-662.67 (0.00)
NMM, Full	1158.12 (13.57)	1588.43 (1.09)	1509 (51.27)
NMM, Red	-15911.88 (214.35)	1330.18 (5.50)	477.96 (541.59)

AIS estimates from *I* = 32 samples and *J* = 512 trajectories. The results for the linear model here* are for the analytic solution, which also corresponds to the VL solution. Entries shows the mean values from 20 runs of each algorithm with standard deviations shown in brackets. ‘App’ denotes the Approach model and ‘NMM’ the two-region neural mass model.

**Table 4:** RMSE between Posterior Mean and True Parameters for Full Model (Multiple Runs).

Model	VL	AIS	AMC4
Linear	0.57 *	0.58 (0.04)	0.57 (0.05)
App	0.015 (0.004)	0.013 (0.008)	0.016 (0.003)
NMM	0.21 (0.00)	0.11 (0.01)	0.38 (0.13)

AIS estimates from *I* = 32 samples and *J* = 512 trajectories. The results for the linear model here* are for the analytic solution, which also corresponds to the VL solution. Entries shows the mean values from 20 runs of each algorithm with standard deviations shown in brackets. ‘App’ denotes the Approach model and ‘NMM’ the two-region neural mass model.

The VL estimates of LogBF for the neural mass model have very low standard deviation, a point which we will comment on further in the next subsection. The corresponding AIS estimates have a standard deviation (or Monte Carlo error) of 5.27. However, this is a small proportion of the absolute value of 271.86. For smaller Bayes factors we expect the AIS Monte Carlo error to be commensurately smaller [13]. This was confirmed by running AIS ten times on the same neural mass models, but with data (additive noise) chosen to produce a signal to noise ratio of unity (see next section). The mean Log Bayes Factor was 11.14 with a standard deviation of 0.66.

To provide an indication as to what level of performance other MCMC methods can provide, we also implemented an Adaptive Monte Carlo (AMC) approach which has been applied to related problems [39, 41]. Specifically we implemented “Algorithm 4” in [42] (which we refer to as AMC4) and collected 2000 samples. The proposal density was adapted for the first 600 samples, and these were then discarded as burn-in. The remaining 1400 samples provided the estimate of the posterior density and were used to compute the model evidence using the Posterior Harmonic Mean (PHM) method (see equation 9 in S1 Text). As the PHM is known to overestimate the model evidence we also implemented Chib’s method [43]. This uses samples from the posterior density and an additional set of samples produced by applying the proposal density to a chosen parameter vector (e.g. posterior mean). For completeness, this is described in S5 Text.

Whilst AMC4 worked well for the linear and approach models (with PHM overestimating the model evidence, as expected) it does not work so well for the neural mass model, as the model evidence approximations are highly variable.

Although AMC4 was run with a modest number of samples this was the same number as in [39], and results were not improved by running the algorithm for longer (we tried collecting 38,000 samples with 3,000 adaption/burn-in). Moreover, we implemented another AMC approach which had two separate phases of adaption (i) tuning of a global scaling parameter for 300 samples, to ensure acceptance rates of between 20 and 40 percent, (ii) tuning of proposal covariance for 300 samples using updates in [44]. Results were again not improved.

#### Effect of signal to noise ratio

The results presented so far have been found in a very high Signal to Noise (SNR) regime, using a very small value for the observation noise standard deviation (SD). Here the SNR is defined as the ratio of the observation noise SD to the signal SD in one of the brain regions (taken arbitrarily to be region 2). So far we have used SNR = 16.

**Fig 8:**
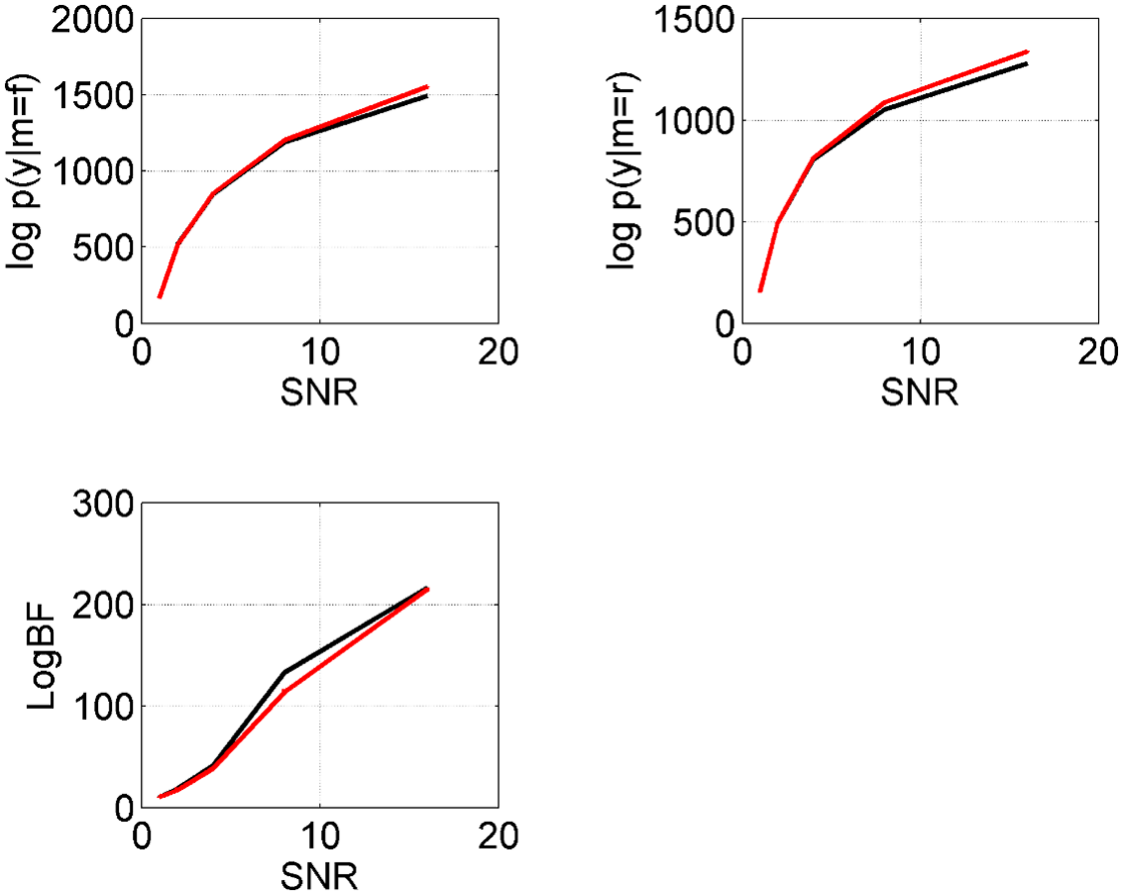
Data from ‘full’ neural mass model: Effect of SNR. Estimates of the log model evidence for full model, log *p*(*y*|*m* = *f*), and reduced model, log *p*(*y*|*m* = *r*), and Bayes factors for full versus reduced for VL (black) and AIS (red) over a range of SNRs.

**Fig 9:**
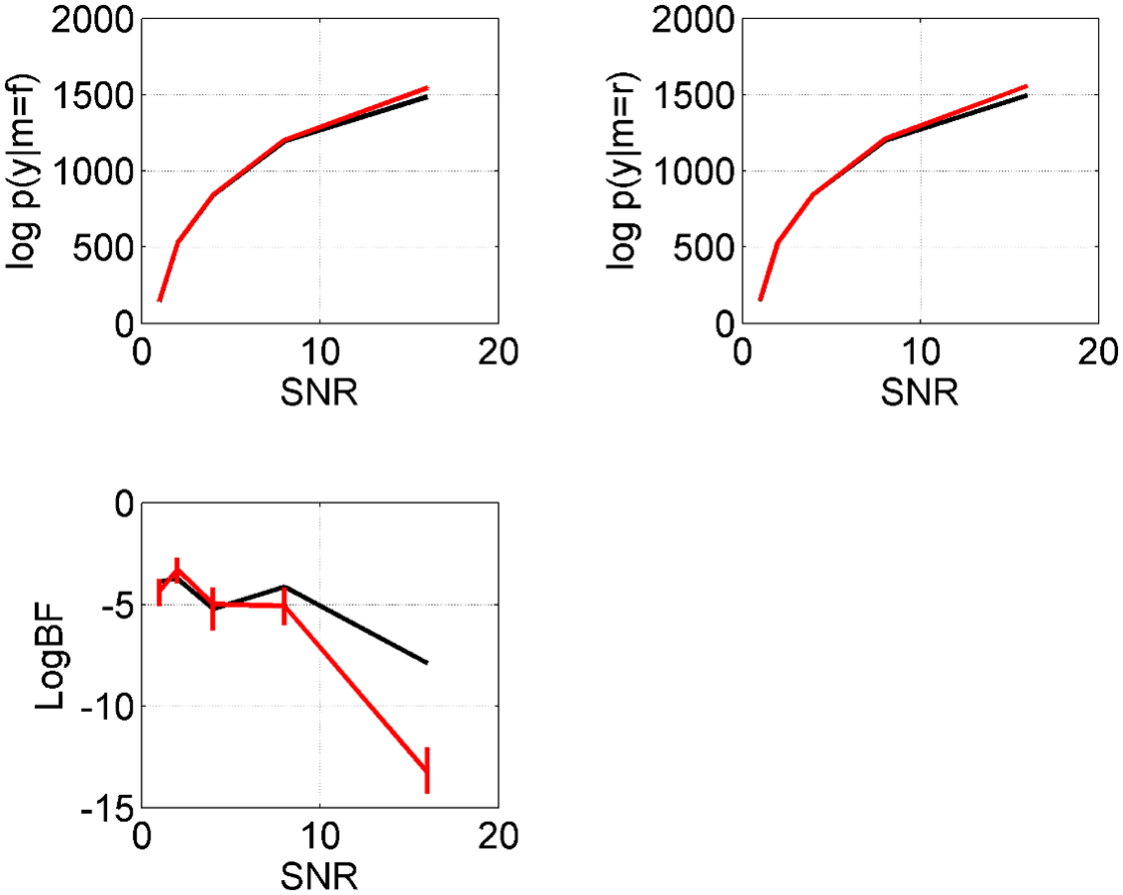
Data from ‘reduced’ neural mass model: Effect of SNR. Estimates of the log model evidence for full model, log *p*(*y*|*m* = *f*), and reduced model, log *p*(*y*|*m* = *r*), and Bayes factors for full versus reduced for VL (black) and AIS (red) over a range of SNRs.

Figs 8 and 9 show results for simulations in which the SNR was varied over a broad range. The results indicate that VL and AIS are generally in agreement, with monotonically increasing estimates of the log evidence as a function of SNR. For both VL and AIS, the log Bayes factors in favour of the full model are increasingly positive with data generated from the full model, and (generally) increasingly negative with data from the reduced model.

There are a number of discrepancies, however, with larger disagreements at high SNR. Overall, AIS tends to produce higher estimates of the log evidence. This is shown more clearly in S1 and S2 Figs for data generated from the full model. ISVL estimates of the model evidence (obtained using 10,000 samples) are also on the high side but have large error bars. Additionally, the ISVL estimates for the reduced model at high SNR were roughly 1000 or more less than for AIS/VL and had huge error bars, so were not plotted on the same figure. We therefore conclude that ISVL is unreliable.

At low SNRs the AIS acceptance rates, *a*_*j*_, averaged over the *I* = 32 trajectories, were relatively constant over *β*_*j*_ whereas for high SNRs there was a gradual decrease with *β*_*j*_. For example, at SNR = 2, *a*_*high*_ = 0.53 and *a*_*low*_ = 0.48 whereas at SNR = 8, *a*_*high*_ = 0.49 and *a*_*low*_ = 0.34.

**Fig 10:**
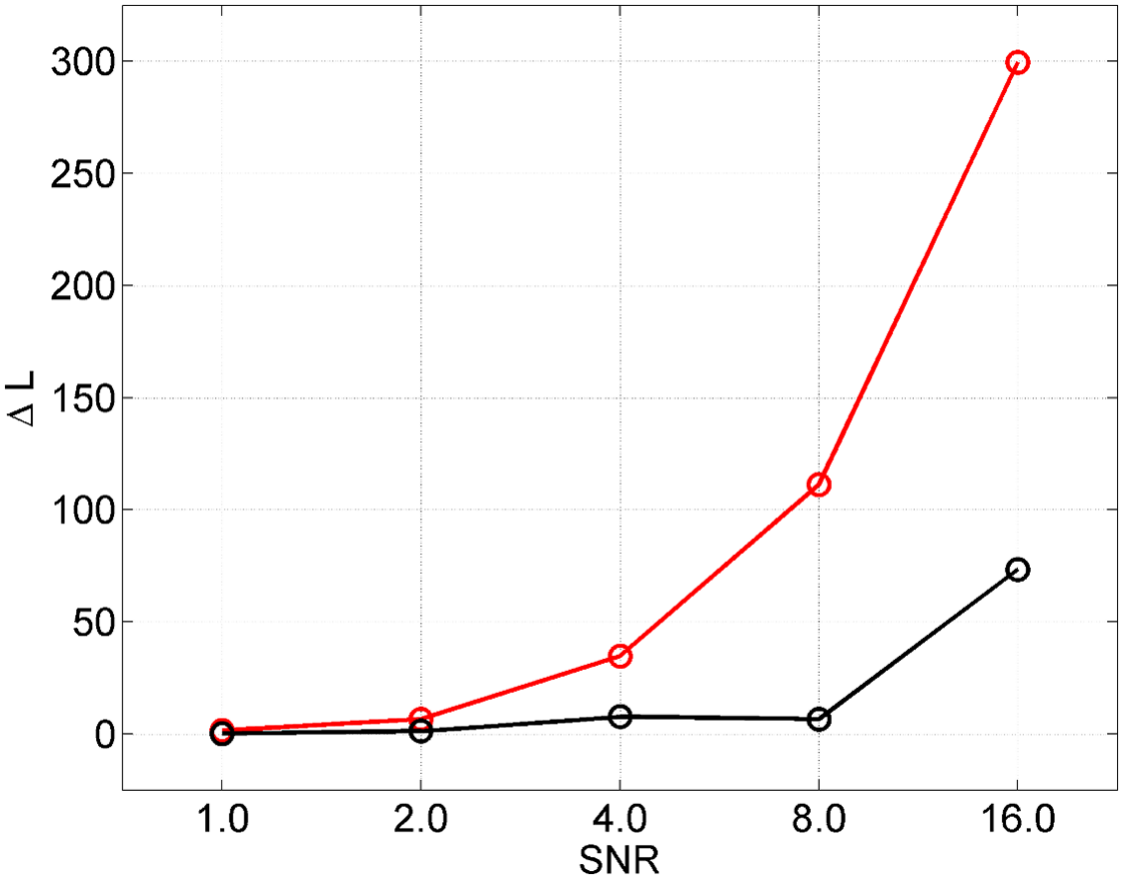
Data from ‘full’ neural mass model: AIS versus Multistart VL. The red curve plots the difference in log joint of the posterior mean from AIS versus that from VL, *L*_*AIS*_ − *L*_*VL*_, and the black curve plots the difference in log joint for the best multistart VL versus VL, *L*_*MVL*_ − *L*_*VL*_.

As earlier, our AIS posterior means tend to have higher log joint probability, *L*, than those from VL. This is demonstrated in Fig 10 which plots the increase in *L* (over baseline VL) as a function of SNR. Here our baseline VL result uses the standard approach of initialisation with the prior mean. A multistart VL approach, however, can also produce better solutions. We used 240 multistarts as before. The maximum number of VL iterations over all multistarts was 42 (which did not exceed our maximal number of 128) and this individual model fit took 58s. Fig 10 plots the improvement offered by the best Multistart VL solution over the standard one showing, for example, an increase of Δ*L* = 73 at the highest SNR. Overall, however, we find the improvement offered by AIS to be superior, with an increase of Δ*L* = 300 at the highest SNR.

Perhaps surprisingly, there was hardly any improvement (or variation) in estimates of the VL model evidence or Bayes Factors over multistarts. As shown in S4 Fig, the variations in Log Bayes Factors are no greater than 0.1 (Bayes Factor = 1.1). Thus, for this neural mass model, VL model inferences show no meaningful variation over multistarts (according to [45] Bayes factors of less than 3 are ‘barely worth a mention’). This is to be contrasted with large variations in the posterior mean over parameters (which led to the improvements in Multistart over baseline VL in Fig 10—see also S3 Fig). This result can perhaps be understood by noting that the model evidence approximation is the cost function that is optimised by VL, as contrasted to more standard Laplace approaches which find parameters that maximise the log joint.

In our initial (high SNR) comparison of AIS and VL estimates of model evidence the discrepancy was larger for the full than for the reduced model (see Fig 6). This did not translate, however, into an incorrect sign in the resulting log Bayes factor as the difference between full and reduced model evidences dominated. A potential concern therefore is that when the model evidences of two models are more similar, errors in evidence estimates will result in errors in log Bayes factors that could produce radically different inferences. However, examples of when model evidences are more similar are provided in the low SNR regimes in Figs 8 and 9. Fig 8, for example, shows that even at the lowest SNR VL agrees with AIS in correctly favouring the more complex model. To examine this further we repeated the simulations at even lower SNR. The results in S5 Fig show, reassuringly, that as the SNR reduces to zero so does the log Bayes Factor (it does not become negative); VL Bayes Factors therefore do not show a bias towards simpler models. Additionally, the VL algorithm exhibits similar behaviour in the context of DCM for fMRI (see eg. Fig. 2 in [37]).

The p-values from Royston’s tests for the various data sets are provided in S2 and S3 Tables. Results are provided for both 32 and 64 AIS trajectories. Most conservatively, considering the 40 multiple statistical comparisons a Bonferroni-corrected p-value of 1.25 × 10^−3^ or less would be seen as significant at the nominal 0.05 level. Given this threshold there is one significantly non-Gaussian posterior distribution. More descriptively, seven of the ten tests with 64 trajectories on data from the full model (two rightmost columns of S2 Table) have p-values of less than 0.05. We can therefore summarise these results by saying we have evidence for non-Gaussianity.

## Discussion

Annealed Importance Sampling has a number of appealing properties. It can provide accurate estimates of the posterior parameter distribution and of the model evidence by avoiding local maxima and without making assumptions of Gaussianity. Samples from AIS converge in distribution to the true posterior density. Sub-optimal model evidence approximations [46] based on the Prior Arithmetic Mean (PAM) or Posterior Harmonic Mean (PHM) emerge as special cases of AIS with only two temperatures. Unlike Markov chain Monte Carlo methods, the samples produced are not serially correlated thus making any corrections involving effective sample size unnecessary.

We have described an implementation of AIS using a transition kernel based on an LMC sampler. The use of LMC here is critical as it allows proposals to be made based on local gradient and curvature information. Our empirical results show that the resulting proposals are accepted with probabilities in a desirable range (similar to the target of 20 to 40% in Zhou et al. [38]) even for nonlinear dynamical systems models at low temperature.

We have compared AIS to inferences based on the VL approximation in the context of neural mass models. In terms of the estimation of Bayes factors, the two methods agree as to which model is best but report different degrees of belief, especially at high signal to noise ratio. AIS tends to produce higher model evidence estimates both for optimal and suboptimal models. AIS finds better parameter estimates than does VL, as quantified by the joint log probability, especially in data regimes with high signal to noise ratio. A possible explanation as to the dependence on SNR could be that there are more or deeper local minima at high SNR. Moreover, a multistart VL procedure with computer time matched to AIS does not find better solutions. Additionally, we found evidence of non-Gaussianity in the AIS posteriors. Thus it appears that AIS is useful due to its ability to avoid local maxima, and its ability to characterise non-Gaussian parameter posteriors.

We have also used an Importance Sampling procedure to estimate the model evidence. This method, which we’ve referred to as ISVL, is highly computationally efficient as it uses the posterior from VL as a proposal density, but it proved unreliable. Similarly, other more standard approaches such as AMC worked well on linear and nonlinear regression problems but it was not possible to derive good AMC-based model evidence estimates for neural mass models.

In order to apply AIS one must decide upon an annealing schedule and in this paper we used a 5th-order geometric schedule, discretised using 512 temperatures and explored using 32 trajectories. This proved sufficient over a range of statistical models from linear and nonlinear regression to nonlinear differential equation models. Our empirical work has shown that the required number of temperatures and trajectories did not show a strong dependence on the number of model parameters or model nonlinearity. However, the need to specify the parametric form of the schedule, number of temperatures and trajectories is clearly a weakness of the AIS approach and is an area of ongoing research.

Previous work in this direction has focussed on the Sequential Monte Carlo (SMC) method which can be viewed as a generalisation of AIS. SMC represents probability densities using particles, as in the particle filter, but is applied at a sequence of temperatures rather than to a sequence of temporally ordered data. In particular Zhou et al. [38] have shown how SMC can be used for model comparison. Automatic annealing schedules can be derived by resampling at every temperature so as to maximise the effective sample size of the particle ensemble. An alternative approach grounded in statistical physics is based on the notion of contact flows and thermodynamic processes [47].

A potential drawback of SMC as compared to AIS, however, is that because particles interact during optimisation, SMC is not amenable to embarrasing parallelisation. Additionally, an application of SMC to nonlinear differential equations [38] used a similar number of temperatures as we do (500 as compared to our 512) but used many more trajectories (1000 as compared to our 32). This suggests that SMC may be more computationally demanding. Another development in this direction is Langevin Importance Sampling [48] which does not require specification of an annealing schedule as temperatures are sampled using Langevin dynamics. This flexibility again comes at the cost of interaction among trajectories (or particles) and therefore also compromises parallelisation.

Beal [23] has also suggested interesting ways of improving AIS. First, automatic annealing schedules could be produced by introducing finer graining of temperatures in regions of the path for which forward and reverse estimates are inconsistent. Second, Eq 11 suggest that better model evidence estimates could be produced by generating more samples at each temperature. This algorithim would then become more similar to thermodynamic integration [46] which, however, is naturally more computationally demanding than AIS [24].

Whilst our model fitting using AIS was parallelised over multiple cores, alternative efforts can be made to speed up implementation. For example, Wang et al. [49] have shown how the integration of neural mass models can be implemented on Graphical Processing Units (GPUs), resulting in a reduction of computing time by a factor of approximately seven. Additionally, Aponte et al. [50] have pursued a similar GPU approach for DCM for fMRI and shown how it can be used in the context of model evidence computation using thermodynamic integration. This GPU approach has been used to estimate parameters of DCM for fMRI models using an Adaptive Monte Carlo algorithm, again resulting in an order of magnitude reduction in computation time [41]. See also [51] for generic methods for parallelisation of single Markov chains.

Dynamical models have also been fitted to neuroimaging data using a range of global optimisation methods. For example, mean field models have been fitted to EEG using particle swarm optimisation [52] and stochastic nonlinear oscillator models have been fitted to EEG using a multi-start algorithm [53]. Additionally, DCMs have been fitted to fMRI data using a method that combines local search with Gaussian process approximation [41]. This method provides better parameter estimates than VL with only a modest increase in computational cost (much less than AIS). However, like the other global optimisation methods (see also [54]), it does not produce an estimate of the posterior distribution or model evidence.

This paper has compared the ability of VL and AIS to make inferences about two-region neural mass models based on simulated data. These simulations are a caricature of the DCM for ERP approach [17] as they are simplified in a number of respects (i) we have fixed parameters such as time delays between regions, synaptic time constants and synaptic response magnitudes, to known true values, (ii) we have not estimated observation noise, (iii) we have used only two brain regions whereas most practical applications use upwards of four [55–57], (iv) we have assumed that the electrical activities of brain regions are directly observed, rather than being filtered through a lead field matrix to produce observations in M/EEG sensor space, (v) we have used simulated rather than empirical M/EEG data. Further work will be needed to establish whether the findings from our caricature follow over to DCM for ERP.

This paper has used an independent model optimisation approach to compute Bayes factors, in which the evidence is computed separately for each model of interest. But in the context of AIS one can traverse a path from the posterior of one model to the posterior of another, with the resulting importance weights providing a direct approximation of the corresponding Bayes factor [13]. Direct computation of Bayes factors in this way is also possible in the context of SMC and a transdimensional AIS algorithm [58]. If one has a nested model, as in the empirical NMM examples in this paper in which the reduced model is nested within the full model model, Savage-Dickey approximations can also be used [59]. It would be interesting to compare Savage-Dickey against the direct path integral methods based on AIS.

This paper has explored one method for combining VL and sampling methods, ISVL, in which the VL posterior is used as a proposal density for importance sampling. However, this method did not provide good estimates of the model evidence. Other proposals for combining sampling with variational methods view the sequence of samples produced by a Markov chain as auxiliary variables in a variational inference problem [60]. An alternative approach, proposed in [13] would be to use AIS to traverse a path from the VL posterior to the true posterior at a series of intermediate temperatures, another interesting avenue for future work.

## Supporting Information

S1 Text Importance sampling. (PDF)

S2 Text Fisher information. (PDF)

S3 Text Neural mass models. (PDF)

S4 Text Variational laplace. (PDF)

S5 Text Chib’s estimate of model evidence. (PDF)

S1 Fig Model evidence estimates for neural mass model: Low SNR. Estimates of the log model evidence for full model, log *p*(*y*|*m* = *f*), and reduced model, log *p*(*y*|*m* = *r*), at low SNR. Vertical lines indicate 95% confidence intervals.
(TIF)

S2 Fig Model evidence estimates for neural mass model: High SNR. Estimates of the log model evidence for full model, log *p*(*y*|*m* = *f*), and reduced model, log *p*(*y*|*m* = *r*), at high SNR. Vertical lines indicate 95% confidence intervals.
(TIF)

S3 Fig VL estimates of log joint over multiple restarts. Estimates of the log joint for full model, log *p*(*y*|*m* = *f*), over multiple restarts and range of SNRs. The baseline VL value (initialisation from prior mean) is shown in red.
(TIF)

S4 Fig VL estimates of Log Bayes Factors over multiple restarts. Estimates of the Log Bayes Factor for full versus reduced models, over multiple restarts and range of SNRs. Data was generated from the full model. The baseline VL value (initialisation from prior mean) is shown in red.
(TIF)

S5 Fig VL estimates of Log Bayes Factors at very low SNR. VL estimates of the Log Bayes Factor for full versus reduced models in very low SNR regime. Data was generated from the full model and the graph plots the mean and 95% confidence intervals computed over 5 data realisations at each SNR.
(TIF)

S1 Table Neural mass model: Parameter transformations. (PDF)

S2 Table Neural mass model: Gaussianity tests on full models. (PDF)

S3 Table Neural mass model: Gaussianity tests on reduced models. (PDF)
