# Supplementary material for: Annealed Importance Sampling for Neural Mass Models
Source: PLoS Comput Biol. 2016 Mar 4;12(3):e1004797. doi: 10.1371/journal.pcbi.1004797 (PMC4778905; doi:10.1371/journal.pcbi.1004797)
Supplement: S1 Text — (PDF) [file pcbi.1004797.s001.pdf]

S1 Text: Importance Sampling

Will Penny*, Biswa Sengupta

Wellcome Trust Centre for Neuroimaging, University College, London, UK

* w.penny@ucl.ac.uk

Importance Sampling

Importance sampling is a generic method for computing the expectation of a function when samples of that function cannot be drawn directly [1]. For example, the expectation of a function $a(w)$ over the density $f(w)/Z_f$ is defined as

$$\bar{a} = \int a(w) \frac{f(w)}{Z_f} dw \tag{1}$$

Multipling the integrand top and bottom by a 'proposal' density $g(w)/Z_g$ and re-arranging gives

$$\bar{a} = \frac{Z_g}{Z_f} \int v(w) a(w) \frac{g(w)}{Z_g} dw \tag{2}$$

where the importance weight $v(w) = f(w)/g(w)$. A Monte Carlo estimate is given by

$$\bar{a} = \frac{Z_g}{Z_f} \frac{1}{I} \sum_{i=1}^{I} v(w_i) a(w_i) \tag{3}$$

where the samples w_i are drawn from the proposal. We can see that

$$\begin{aligned} \bar{v} &= \frac{1}{I} \sum_i v(w_i) \\ &= \frac{Z_f}{Z_g} \end{aligned} \tag{4}$$

Hence $\sum_i v(i) = I Z_f / Z_g$. We can therefore write

$$\bar{a} = \frac{\sum_i v(w_i) a(w_i)}{\sum_i v(w_i)} \tag{5}$$

Model Evidence

By letting $a(w) = p(y|w, m)$ and $f(w) = p(w)$ we have

$$\begin{aligned} a(w) &= \int p(y|w, m) p(w) dw \\ &= p(y|m) \end{aligned} \tag{6}$$

An importance sampling estimate of the model evidence is therefore given by

$$
\begin{aligned}
p_{IS}(y|m) &= \frac{\sum_{i=1}^{I} v^{(i)} p(y|w_i, m)}{\sum_{i=1}^{I} v^{(i)}} \\
v^{(i)} &= \frac{p(w_i|m)}{q(w_i|m)}
\end{aligned}
\tag{7}
$$

where q is known as an importance or approximating density (previously g/Z_g), w_i are samples from q, and $v^{(i)}$ are referred to as importance weights. Different choices for q give rise to different IS approximations to the model evidence.

The simplest choice is the prior density, $q(w|m) = p(w|m)$, which gives rise to the Prior Arithmetic Mean

$$
p_{PAM}(y|m) = \frac{1}{I} \sum_{i=1}^{I} p(y|w_i, m) \tag{8}
$$

This approximation can of course be motivated from a simple Monte Carlo approximation to the evidence integral. A problem with this estimate, however, is that most samples from the prior will have low likelihood. A large number of samples will therefore be required to ensure that high likelihood regions of parameter space will be included in the average. If this does not occur then the model evidence will be under-estimated.

A second choice is the posterior density, $q(w|m) = p(w|y, m)$. Application of Bayes rule to the numerator and denominator of equation 7 then leads to the expression for the Posterior Harmonic Mean

$$
p_{PHM}(y|m) = \left[\frac{1}{I} \sum_{i=1}^{I} \frac{1}{p(y|w_i, m)} \right]^{-1} \tag{9}
$$

A problem with the PHM is that the largest contributions come from low likelihood samples which results in a high-variance estimator. In applications to phylogenetic networks, the PHM has been shown to overestimate the model evidence [2].

A third possibility which we explore in this paper is p_{ISVL} which uses equation 7 with a proposal density given by the posterior from VL optimisation. Being a Gaussian this is straightforward to sample from and the importance weights are given by the ratio of the probability of the sample under the prior versus under the VL posterior.

Reverse Annealing

By inverting the equation for the model evidence we have

$$
\begin{aligned}
\frac{1}{p(y|m)} &= \frac{Z_1}{Z_J} \\
&= \frac{Z_{J-1}}{Z_J} \cdots \frac{Z_2}{Z_3} \cdots \frac{Z_1}{Z_2} \\
&= \prod_{j=1}^{J-1} \frac{1}{r_j}
\end{aligned}
\tag{10}
$$

For $J = 2$ temperatures $\beta_2 = 1$, $\beta_1 = 0$ we get

$$
\frac{1}{p(y|m)} = \frac{1}{p(y|w, m)} \tag{11}
$$

Averaging over multiple trajectories gives

$$\frac{1}{p(y|m)} = \frac{1}{I} \sum_{i=1}^{I} \frac{1}{p(y|w_i, m)} \tag{12}$$

which shows that the PHM approximation to the model evidence is a special case of AIS with a reverse annealing schedule and only $J = 2$ temperatures. Importance weights for reverse annealing are given by

$$v^{(i)} = \frac{f_{J-1}(w_{J-1})}{f_J(w_{J-1})} ... \frac{f_2(w_2)}{f_3(w_2)} \frac{f_1(w_1)}{f_2(w_1)} \tag{13}$$

and a series of samples $w_J, w_{J-1}, ...w_2, w_1$ are created by starting with w_J from forward annealing, and generating the others sequentially using LMC.

Importance Weights

To derive the importance weights for AIS we consider the forward and backward joint densities over the whole trajectory. The backward density constitutes our target f and the forward constitutes our proposal g. If the importance weights are chosen to correct for discrepancies between them, then expectations based on $w_1, ..., w_J$ will be correct. If expectations over the joint density are correct then so will be those over any element of the joint. Thus w_J will be a sample from the posterior density. The density of the backward sequence is

$$f(w_1, ..., w_J) = f_J(w_J) \tilde{T}_{J-1}(w_{J-1}|w_J) ... \tilde{T}_1(w_1|w_2) \tag{14}$$

where the backward transition kernel is related to the forward as

$$\tilde{T}_j(w_j|w_{j+1}) = T_j(w_{j+1}|w_j) \frac{f_j(w_j)}{f_j(w_{j+1})} \tag{15}$$

We can therefore write

$$f(w_1, ..., w_J) = \frac{f_J(w_J)}{f_{J-1}(w_J)} ... \frac{f_2(w_2)}{f_1(w_2)} f_1(w_1) \prod_{j=1}^{J-1} T_j(w_{j+1}|w_j) \tag{16}$$

The density of the forward sequence is

$$g(w_1, ..., w_J) = f_0(w_1) \prod_{j=1}^{J-1} T_j(w_{j+1}|w_j) \tag{17}$$

Hence the importance weights are given by

$$v = \frac{f(w_1, ..., w_J)}{g(w_1, ..., w_J)} \tag{18}$$

which is equal to

$$v = \frac{f_1(w_1)}{f_0(w_1)} \frac{f_2(w_2)}{f_1(w_2)} ... \frac{f_J(w_J)}{f_{J-1}(w_J)} \tag{19}$$

Because the mean importance weight is equal to Z_f/Z_g (from equation 4), then if f_0 is the prior and f_J is the unnormalised posterior, the mean importance weight is also equal to the model evidence [3].

References

1. Mackay DJC. Information Theory, Inference and Learning Algorithms. Cambridge University Press, Cambridge; 2003.
2. Lartillot N, Philippe H. Computing Bayes factors using thermodynamic integration. Systematic Biology. 2006;55(2):195–207.
3. Neal RM. Annealed Importance Sampling. Statistics and Computing. 2001;11:125–139.
