# Supplementary material for: Annealed Importance Sampling for Neural Mass Models
Source: PLoS Comput Biol. 2016 Mar 4;12(3):e1004797. doi: 10.1371/journal.pcbi.1004797 (PMC4778905; doi:10.1371/journal.pcbi.1004797)
Supplement: S2 Text — (PDF) [file pcbi.1004797.s002.pdf]

S2 Text: Fisher Information

Will Penny*, Biswa Sengupta

Wellcome Trust Centre for Neuroimaging, University College, London, UK

* w.penny@ucl.ac.uk

Fisher Information

If the log likelihood is $R = \log p(y|w)$ then the Fisher score is the gradient

$$g = \frac{\partial R}{\partial w} \tag{1}$$

The Fisher information matrix is then the covariance of the score

$$\begin{aligned} F &= \mathsf{Cov}\,[g] \\ &= E_{p(y|w)}\left[gg^T\right] \end{aligned} \tag{2}$$

where the second line follows as the expected score is zero.

For multivariate Gaussian likelihoods (with precision Γ) about predictions $f(t)$ we have

$$\begin{aligned} g(t) &= S_t^T \Gamma [y(t) - f(t)] \\ g &= \sum_{t=1}^{T} g(t) \end{aligned} \tag{3}$$

where t indexes the observation, and S_t is the sensitivity matrix, or derivative of the predictions with respect to the parameters. The Fisher Information can then be computed by taking expectations, giving

$$F = \sum_{t=1}^{T} S_t^T \Gamma S_t \tag{4}$$

A sample-based or 'observed' Fisher information matrix can be computed as

$$F^{obs} = \sum_{t=1}^{T} g(t) g(t)^T \tag{5}$$

The Fisher Information matrix can also be written as the expected curvature of the likelihood

$$F = -E_{p(y|w)}\left[\frac{\partial^2 R}{\partial w^2}\right] \tag{6}$$

A general expression for the observed Fisher Information [1] is

$$F^{obs} = -H \tag{7}$$

where H is the Hessian (matrix of second order partial derivatives) of R. This can be evaluated numerically and is used when analytic expressions for F are unavailable. A drawback of this approach is that it is expensive computationally, with evaluation time being quadratic in the number of model parameters. A concern with the observed Fisher Information is that it is not necessarily positive definite, however, this is ameliorated in a Bayesian setting where the prior precision is added to it before matrix inversion.

References

1. Efron B, Hinkley D. Assessing the accuracy of the maximum likelihood estimator: Observed versus expected Fisher Information. Biometrika. 1978;65.
