# Supplementary material for: Annealed Importance Sampling for Neural Mass Models
Source: PLoS Comput Biol. 2016 Mar 4;12(3):e1004797. doi: 10.1371/journal.pcbi.1004797 (PMC4778905; doi:10.1371/journal.pcbi.1004797)
Supplement: S3 Text — (PDF) [file pcbi.1004797.s003.pdf]

S3 Text: Neural Mass Models

Will Penny*, Biswa Sengupta

Wellcome Trust Centre for Neuroimaging, University College, London, UK

* w.penny@ucl.ac.uk

Neural Mass Models

The equations for the first cortical region are

$$\begin{aligned} v_i(1) &= [a_{12}s(\bar{v}_p(2)) + \gamma_3 s(\tilde{v}_p(1))] \otimes h_e \\ v_s(1) &= [u + \gamma_1 s(\tilde{v}_p(1))] \otimes h_e \\ v_{pe}(1) &= [a_{12}s(\bar{v}_p(2)) + \gamma_2 s(\tilde{v}_s(1))] \otimes h_e \\ v_{pi}(1) &= \gamma_4 s(\tilde{v}_i(1)) \otimes h_i \\ v_p(1) &= v_{pe}(1) - v_{pi}(1) \end{aligned} \quad (1)$$

and for the second region are

$$\begin{aligned} v_i(2) &= \gamma_3 s(\tilde{v}_p(2)) \otimes h_e \\ v_s(2) &= [a_{21}s(\bar{v}_p(1)) + \gamma_1 s(\tilde{v}_p(2))] \otimes h_e \\ v_{pe}(2) &= \gamma_2 s(\tilde{v}_s(2)) \otimes h_e \\ v_{pi}(2) &= \gamma_4 s(\tilde{v}_i(2)) \otimes h_i \\ v_p(2) &= v_{pe}(2) - v_{pi}(2) \end{aligned} \quad (2)$$

Parameters γ_1 to γ_4 denote within-unit or 'intrinsic' connection strengths. In these equations $\tilde{v}$ denotes the potential after a delay τ_{ii} due to signalling delays among the different cell populations within a cortical region. Here we use a first order Taylor series, $\tilde{v} = v - \tau_{ii}\dot{v}$ to capture these within-region (or 'intrinsic') delays. Similarly, $\bar{v} = v - \tau_{ij}\dot{v}$ captures the 'extrinsic' delay, τ_{ij}, from region j to i.

Differential equations

Each synapse

$$v_{out}(t) = h_e(t) \otimes s(v_{in}(t)) \quad (3)$$

$$h_e(t) = \frac{H_e}{\tau_e} t \exp(-t/\tau_e) \quad (4)$$

can be implemented with a second order DE or two first order DEs [1]

$$\dot{v}_{out} = c_{out} \quad (5)$$

$$\dot{c}_{out} = \frac{H_e}{\tau_e} s(v_{in}) - \frac{2}{\tau_e} c_{out} - \frac{1}{\tau_e^2} v_{out} \quad (6)$$

where c_{out} is the current flowing through the synapse. Hence each synapse gives rise to two DEs. The convolution equations that define neural masses then become a set of differential equations. For a single cortical unit we have

$$\begin{aligned}
\dot{v}_s &= c_s \\
\dot{v}_{pe} &= c_{pe} \\
\dot{v}_{pi} &= c_{pi} \\
\dot{c}_s &= \frac{H_e}{\tau_e}\gamma_3(s(u) + \gamma_1 s(v_p) - \frac{2}{\tau_e}c_s - \frac{1}{\tau_e^2}v_s \\
\dot{c}_{pe} &= \frac{H_e}{\tau_e}\gamma_2 s(v_s) - \frac{2}{\tau_e}c_{pe} - \frac{1}{\tau_e^2}v_{pe} \\
\dot{c}_{pi} &= \frac{H_i}{\tau_i}\gamma_4 s(v_i) - \frac{2}{\tau_i}c_{pi} - \frac{1}{\tau_i^2}v_{pi} \\
\dot{v}_i &= c_i \\
\dot{c}_i &= \frac{H_e}{\tau_e}\gamma_3 s(v_p) - \frac{2}{\tau_e}c_i - \frac{1}{\tau_e^2}v_i \\
\dot{v}_p &= c_{pe} - c_{pi}
\end{aligned} \tag{7}$$

References

1. Grimbert F, Faugeras O. Bifurcation analysis of Jansen's neural mass model. Neural Comput. 2006;18(12):3052–68.
