# Supplementary material for: Annealed Importance Sampling for Neural Mass Models
Source: PLoS Comput Biol. 2016 Mar 4;12(3):e1004797. doi: 10.1371/journal.pcbi.1004797 (PMC4778905; doi:10.1371/journal.pcbi.1004797)
Supplement: S4 Text — (PDF) [file pcbi.1004797.s004.pdf]

S4 Text: Variational Laplace

Will Penny*, Biswa Sengupta

Wellcome Trust Centre for Neuroimaging, University College, London, UK

* w.penny@ucl.ac.uk

Variational Laplace

The Variational Laplace (VL) algorithm [1] can be used for Bayesian estimation of any nonlinear model of the form

$$y = f(\theta, m) + e \tag{1}$$

where $f(\theta)$ is a nonlinear function specified by model m, and e is zero mean additive Gaussian noise with covariance C_y. This covariance depends on hyperparameters λ as shown below. The likelihood of the data is therefore

$$p(y|\theta, \lambda, m) = \mathsf{N}(y; f(\theta, m), C_y) \tag{2}$$

The framework allows for Gaussian priors over model parameters

$$p(\theta|m) = \mathsf{N}(\theta; \mu_\theta, C_\theta) \tag{3}$$

where the prior mean and covariance are assumed known. The error covariances are assumed to decompose into terms of the form

$$C_y^{-1} = \sum_i \exp(\lambda_i) Q_i \tag{4}$$

where Q_i are known precision basis functions. The 'noise parameters' or hyperparameters that govern these error precisions are collectively written as the vector λ. These will be estimated. Additionally, the hyperparameters are constrained by the prior

$$p(\lambda|m) = \mathsf{N}(\lambda; \mu_\lambda, C_\lambda) \tag{5}$$

The above distributions allow one to write down an expression for the joint probability of the data, parameters and noise parameters

$$p(y, \theta, \lambda|m) = p(y|\theta, \lambda, m)p(\theta|m)p(\lambda|m) \tag{6}$$

The starting point for variational inference is then to assume, where necessary, a factorisation of the posterior density [2]. The VL algorithm is based on the assumption that the approximate posterior density has the following factorised form

$$\begin{aligned} q(\theta, \lambda|y, m) &= q(\theta|y, m)q(\lambda|y, m) \\ q(\theta|y, m) &= \mathcal{N}(\theta; m_\theta, S_\theta^{-1}) \\ q(\lambda|y, m) &= \mathcal{N}(\lambda; m_\lambda, S_\lambda^{-1}) \end{aligned} \tag{7}$$

where $\mathcal{N}(x; m_x, \Lambda_x)$ denotes a multivariate Gaussian variable x with mean m_x and precision Λ_x. Importantly, the factorisation is between parameters and noise parameters

only. Dependencies among model parameters are explicitly accounted for in the posterior covariance matrix S_θ. For a model with p parameters S_θ is a $[p \times p]$ matrix.

The parameters of the above approximate posteriors are iteratively updated so as to minimise the Kullback-Liebler divergence between the true and approximate posteriors. This algorithm is described fully in [1]. Updates for the noise parameters in the context of MEG source reconstruction are provided in [3]. In the current paper, however, the prior over the noise parameters is exceptionally tight over known true values, such that optimisation of the noise parameters, λ, is redundant.

Model Evidence

The Negative Variational Free Energy is defined as

$$F(m) = \int \int q(\theta|y,m) q(\lambda|y,m) \log \left[\frac{p(y,\theta,\lambda|m)}{q(\theta|y,m)q(\lambda|y,m)} \right] d\theta d\lambda \tag{8}$$

where

$$p(y,\theta,\lambda|m) = p(y|\theta,\lambda,m)p(\theta|m)p(\lambda|m) \tag{9}$$

This quantity provides a lower bound on the log model evidence [2]. As shown in [4, 5] (and equation 21 in [1]) the VL approximation to $F(m)$ is given by

$$\begin{aligned} F_L(m) &= -\frac{1}{2} e_y^T C_y^{-1} e_y - \frac{1}{2} \log |C_y| - \frac{N}{2} \log 2\pi \\ &- \frac{1}{2} e_\theta^T C_\theta^{-1} e_\theta - \frac{1}{2} \log |C_\theta| + \frac{1}{2} \log |S_\theta| \\ &- \frac{1}{2} e_\lambda^T C_\lambda^{-1} e_\lambda - \frac{1}{2} \log |C_\lambda| + \frac{1}{2} \log |S_\lambda| \end{aligned} \tag{10}$$

where N is the number of data points and the error terms are

$$\begin{aligned} e_y &= y - f(m_\theta, m) \\ e_\theta &= m_\theta - \mu_\theta \\ e_\lambda &= m_\lambda - \mu_\lambda \end{aligned} \tag{11}$$

Generically, factorised variational approximations provide a lower bound on the log model evidence [2]. The difference between the true log model evidence and $F(m)$ is given by the Kullback-Liebler divergence between the true and variational posterior. Thus, as this KL divergence increases the bound becomes less tight and $F(m)$ will not provide an accurate approximation. It turns out, however, that F_L provides an *approximation* to the model evidence rather than a lower bound [4, 5] (it can be lower or higher than $F(m)$). Empirically, however, it has been shown to provide a better model selection measure than does AIC or BIC [5]. The quantity $F_L(m)$ is the VL model evidence approximation referred to in the paper.

References

1. Friston K, Mattout J, Trujillo-Barreto N, Ashburner J, Penny W. Variational free energy and the Laplace approximation. Neuroimage. 2007;34(1):220–234.

2. Beal M. Variational Algorithms for Approximate Bayesian Inference. Gatsby Computational Neuroscience Unit, University College London; 2003.

3. Lopez J, Litvak V, Espinosa J, Friston K, Barnes G. Algorithmic procedures for Bayesian MEG/EEG source reconstruction in SPM. Neuroimage. 2014;84:476–487.

4. Wipf D, Nagarajan S. A unified Bayesian framework for MEG/EEG source imaging. Neuroimage. 2009;44(3):947–966.

5. Penny WD. Comparing Dynamic Causal Models using AIC, BIC and Free Energy. Neuroimage. 2011;59(1):319–330.
