# Supplementary material for: Annealed Importance Sampling for Neural Mass Models
Source: PLoS Comput Biol. 2016 Mar 4;12(3):e1004797. doi: 10.1371/journal.pcbi.1004797 (PMC4778905; doi:10.1371/journal.pcbi.1004797)
Supplement: S5 Text — (PDF) [file pcbi.1004797.s005.pdf]

S5 Text: Chib's Estimate of Model Evidence

Will Penny*, Biswa Sengupta

Wellcome Trust Centre for Neuroimaging, University College, London, UK

* w.penny@ucl.ac.uk

Chib's Estimate

Chib's estimate is derived from a rearrangement of Bayes rule, expressing the evidence in terms of the prior, likelihood and posterior, evaluated using parameters $\hat{w}$. That is

$$p(y) = \frac{p(y|\hat{w})p(\hat{w})}{p(\hat{w}|y)} \tag{1}$$

where $\hat{w}$ is chosen to have high probability under the posterior. In our implementation we choose $\hat{w}$ to be the posterior mean. The likelihood and prior probabilities are readily computed at this point leaving only the problematic posterior term $p(\hat{w}|y)$.

If samples were produced using Gibbs Sampling this term can be estimated as described in [1]. If they were produced using a single block Metropolis-Hastings (MH) sampler a method is described in Chib and Jeliazkov [2] (this paper also describes methods for more structured samplers). This latter method can be derived from the requirement of detailed balance

$$p_T(w|\hat{w})p(\hat{w}|y) = p_T(\hat{w}|w)p(w|y) \tag{2}$$

where $p(w|y)$ is the posterior density of model parameters w given data y, and the probability of moving from parameter $\hat{w}$ to w in the Markov Chain is given by

$$p_T(w|\hat{w}) = \alpha(w|\hat{w})p(w|\hat{w}) \tag{3}$$

where $p(w|\hat{w})$ is the proposal density and the MH acceptance probability is given by

$$\alpha(w|\hat{w}) = \min\left[1, \frac{p_w(w)}{p_w(\hat{w})}\frac{p(\hat{w}|w)}{p(w|\hat{w})}\right] \tag{4}$$

where the joint probability $p_w(w) = p(y|w)p(w)$.

Substituting equation 3 into equation 2, integrating over w, and re-arranging gives equation 8 in [2], which in our notation is

$$p(\hat{w}|y) = \frac{\int \alpha(\hat{w}|w)p(\hat{w}|w)p(w|y)dw}{\int \alpha(w|\hat{w})p(w|\hat{w})dw} \tag{5}$$

This is the ratio of two expectations

$$\begin{aligned} p(\hat{w}|y) &= \frac{E_{num}}{E_{denom}} \\ E_{num} &= E_{p(w|y)}\left[\alpha(\hat{w}|w)p(\hat{w}|w)\right] \\ E_{denom} &= E_{p(w|\hat{w})}\left[\alpha(w|\hat{w})\right] \end{aligned} \tag{6}$$

The numerator expectation can be evaluated using samples from the Markov Chain, w_s (as these are from the posterior). That is

$$E_{num} = \frac{1}{S}\sum_{s=1}^{S}\alpha(\hat{w}|w_s)p(\hat{w}|w_s) \tag{7}$$

where S is the number of samples from the chain (after burn-in). If we record the joint probability of samples from the chain then this quantity is readily computed, as only the joint probability at $\hat{w}$ needs to be computed.

The denominator expectation is given by

$$E_{denom} = \frac{1}{J}\sum_{j=1}^{J}\alpha(w_j|\hat{w}) \tag{8}$$

and w_j are samples from $p(w|\hat{w})$. This does not require sampling from a Markov chain, merely from the proposal density centred at $\hat{w}$. It does, however, require J evalutions of the joint probability $p_w(w_j)$. If we are fitting differential equation models we therefore have to integrate our models an additional J times to compute these values. However, J can be less than S.

To summarise, Chib's estimate uses samples already produced by a Markov chain, and an additional set of new samples. This additional set is potentially smaller and is not produced from a chain, but rather from the proposal density centred around e.g. the posterior mean. In our implementation, however, we used $J = S$.

References

1. Chib S. Marginal Likelihood from the Gibbs Output. Journal of the American Statistical Association. 1995;90:1313–1321.

2. Chib S, Jeliazkov I. Marginal Likelihood from the Metropolis-Hastings Output. Journal of the American Statistical Association. 2001;96(453):270–281.
