# Supplementary figures and images for: Annealed Importance Sampling for Neural Mass Models
Source: PLoS Comput Biol. 2016 Mar 4;12(3):e1004797. doi: 10.1371/journal.pcbi.1004797 (PMC4778905; doi:10.1371/journal.pcbi.1004797)

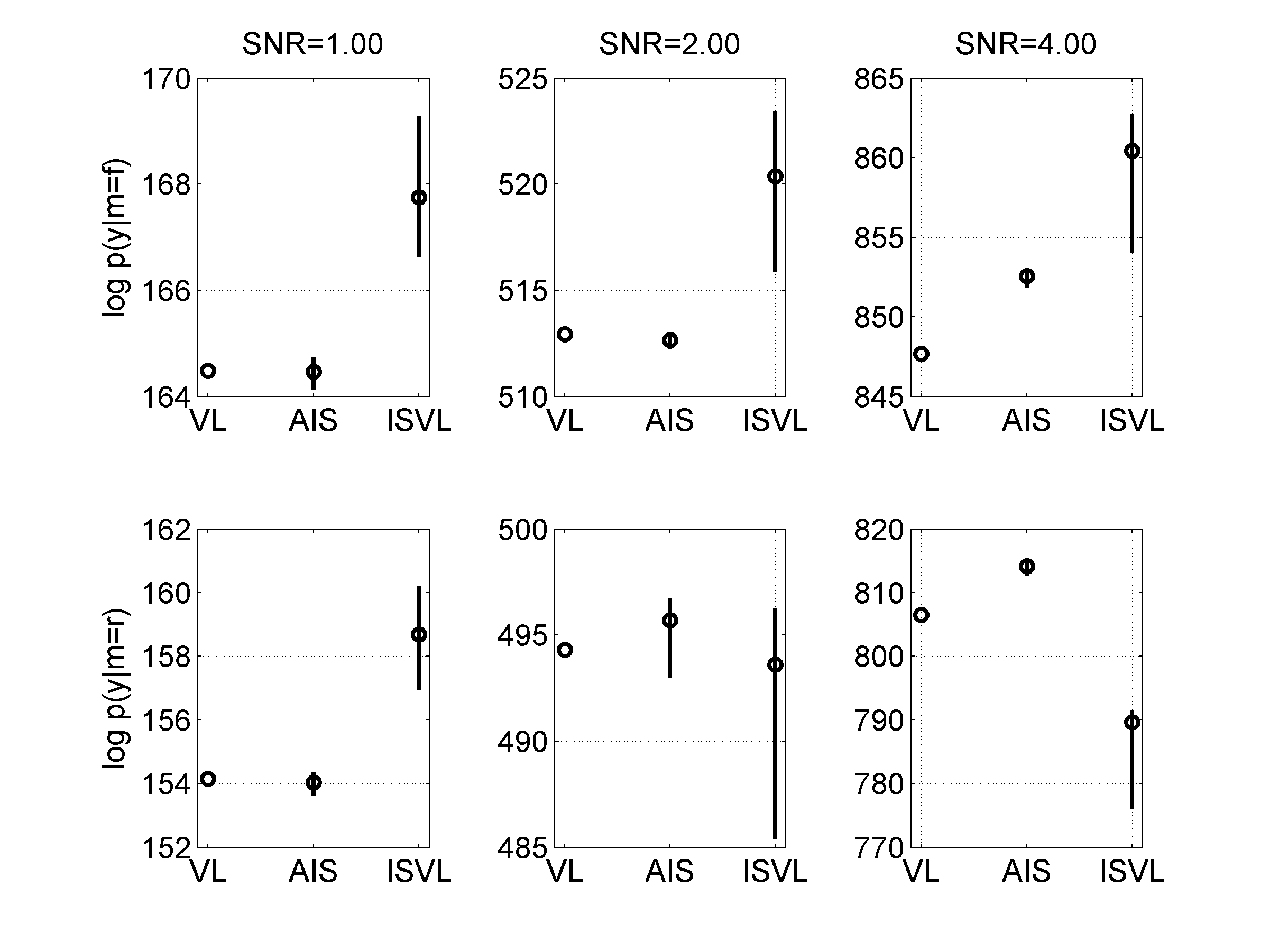

Supplement: S1 Fig — Estimates of the log model evidence for full model, log p(y|m = f), and reduced model, log p(y|m = r), at low SNR. Vertical lines indicate 95% confidence intervals. (TIF) [file pcbi.1004797.s006.tif]

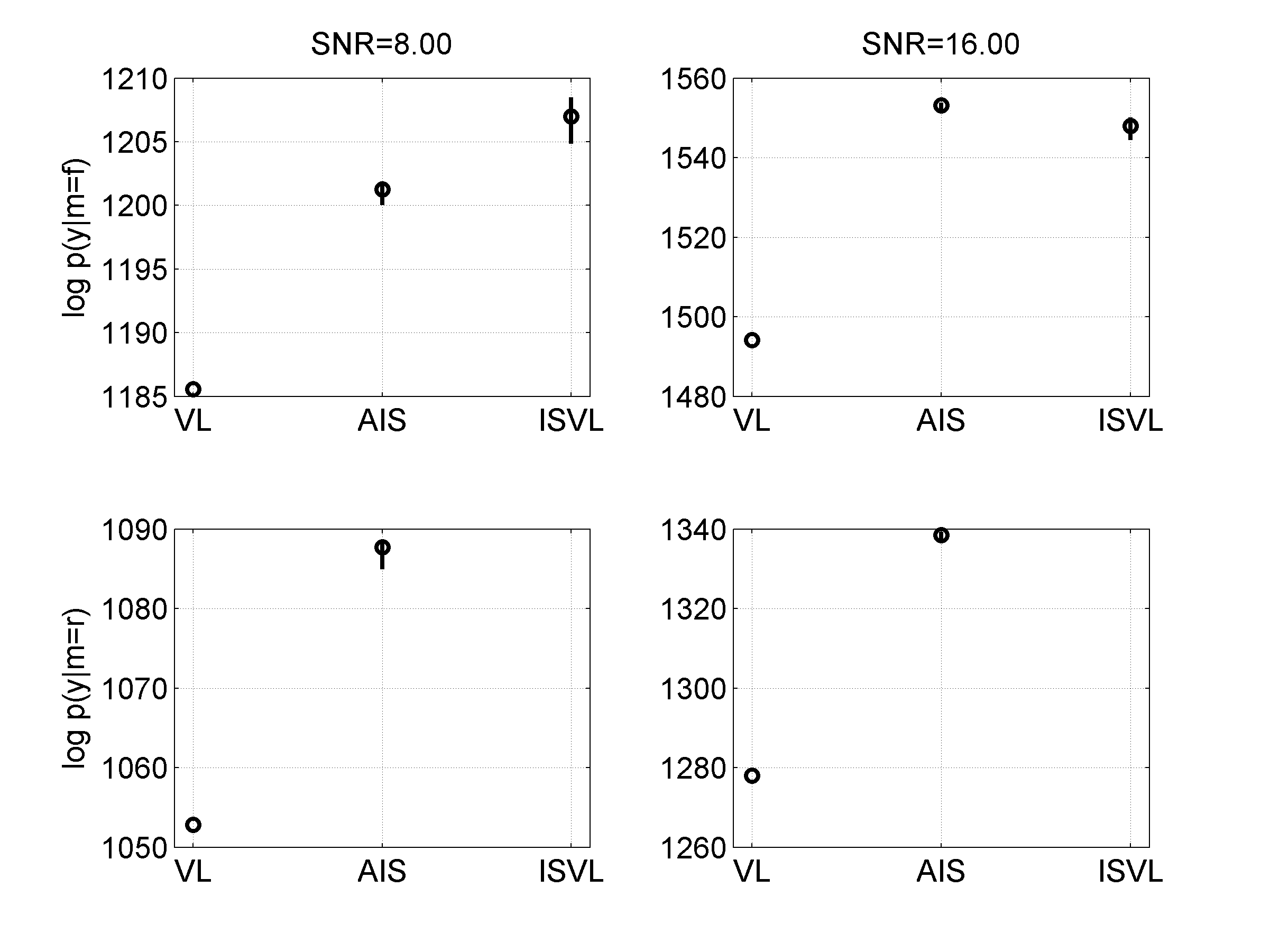

Supplement: S2 Fig — Estimates of the log model evidence for full model, log p(y|m = f), and reduced model, log p(y|m = r), at high SNR. Vertical lines indicate 95% confidence intervals. (TIF) [file pcbi.1004797.s007.tif]

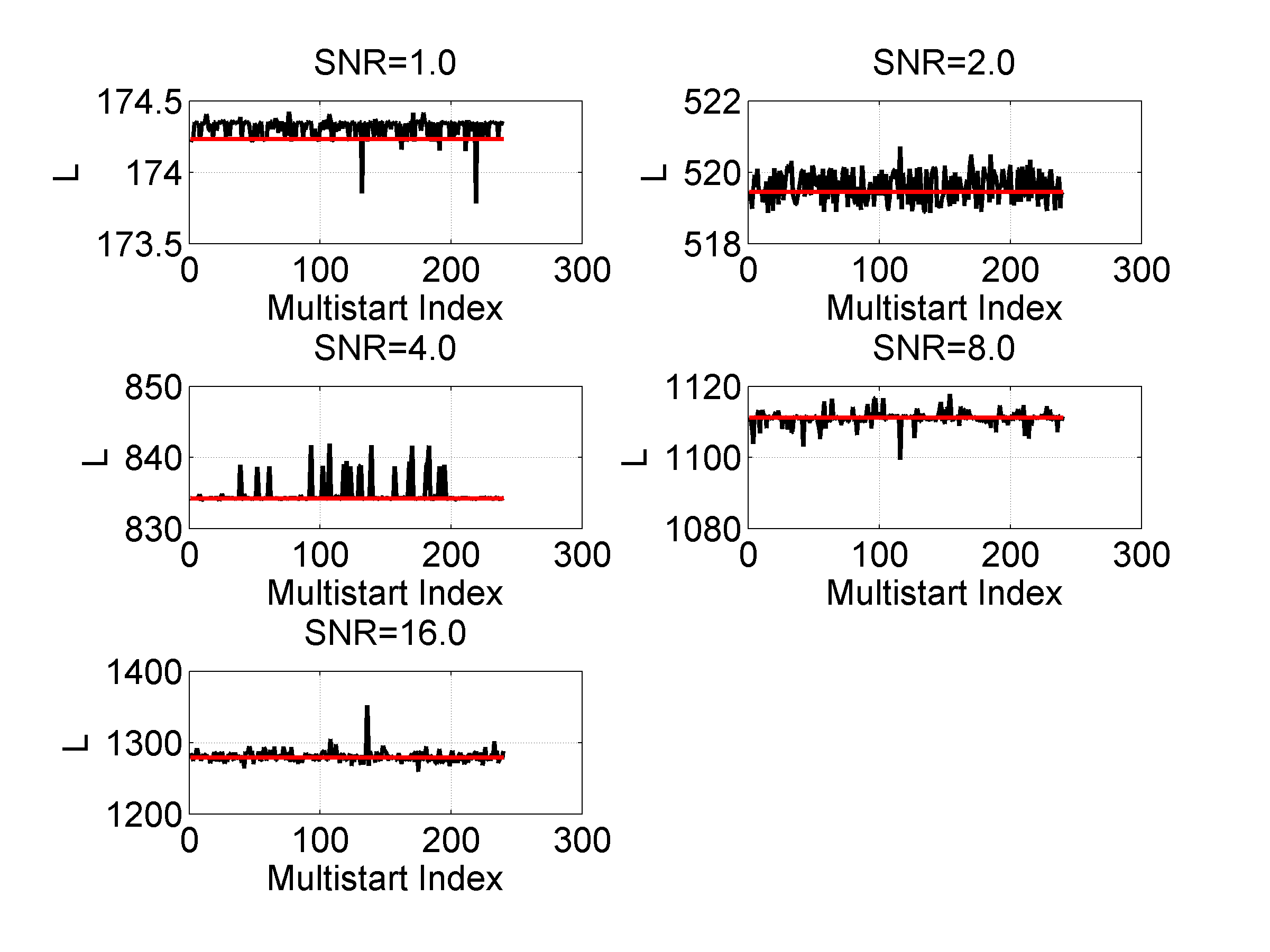

Supplement: S3 Fig — Estimates of the log joint for full model, log p(y|m = f), over multiple restarts and range of SNRs. The baseline VL value (initialisation from prior mean) is shown in red. (TIF) [file pcbi.1004797.s008.tif]

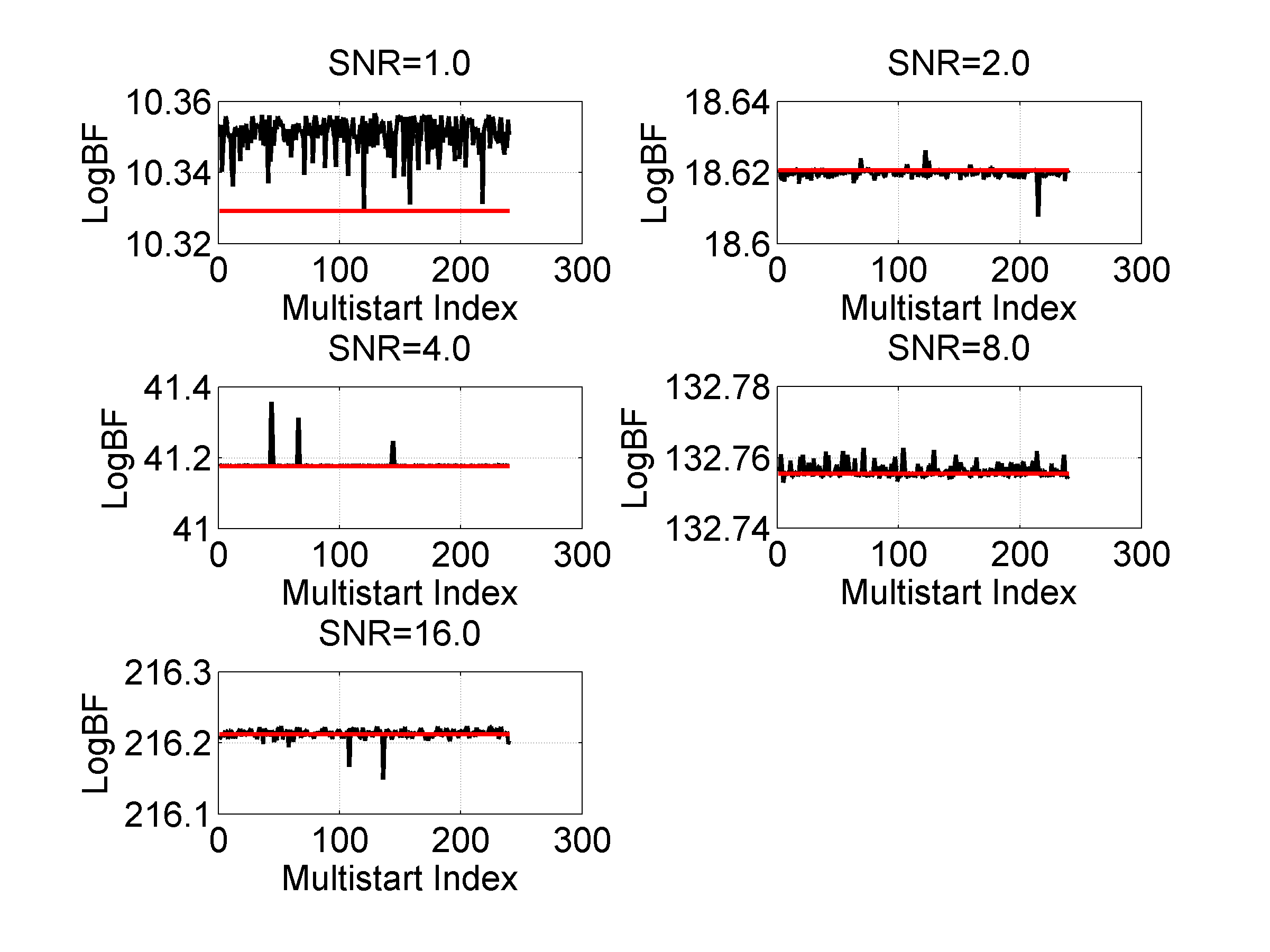

Supplement: S4 Fig — Estimates of the Log Bayes Factor for full versus reduced models, over multiple restarts and range of SNRs. Data was generated from the full model. The baseline VL value (initialisation from prior mean) is shown in red. (TIF) [file pcbi.1004797.s009.tif]

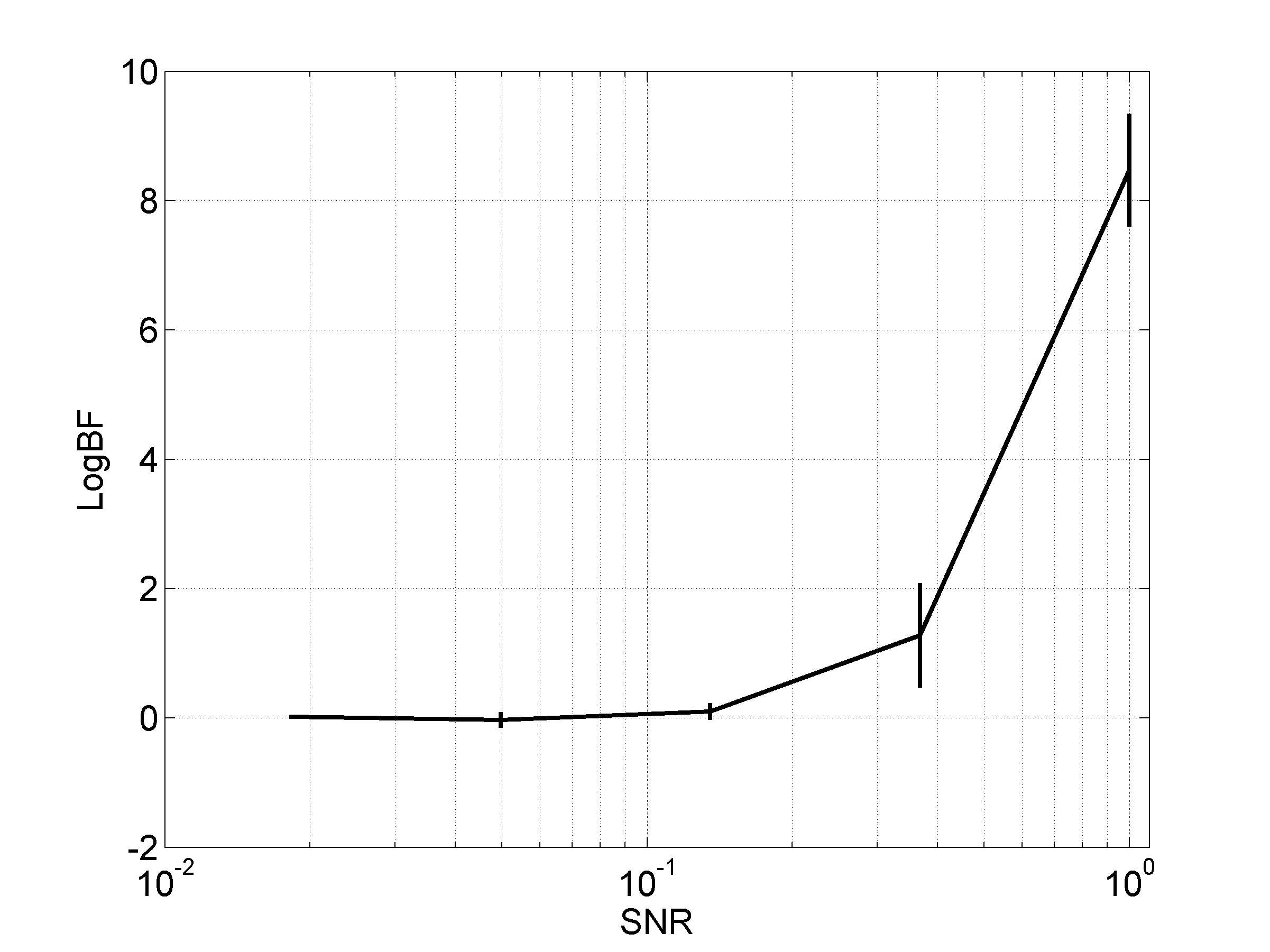

Supplement: S5 Fig — VL estimates of the Log Bayes Factor for full versus reduced models in very low SNR regime. Data was generated from the full model and the graph plots the mean and 95% confidence intervals computed over 5 data realisations at each SNR. (TIF) [file pcbi.1004797.s010.tif]
