# Supplementary material for: Annealed Importance Sampling for Neural Mass Models
Source: PLoS Comput Biol. 2016 Mar 4;12(3):e1004797. doi: 10.1371/journal.pcbi.1004797 (PMC4778905; doi:10.1371/journal.pcbi.1004797)
Supplement: S1 Table — (PDF) [file pcbi.1004797.s011.pdf]

Table 1. Parameter Transformations for Neural Mass Model

$a_{12} = 16\exp(w_1)$	$\gamma_1 = 128\exp(w_5)$
$a_{21} = 32\exp(w_2)$	$\gamma_2 = 102\exp(w_6)$
$\delta_{12} = 16\exp(w_3)$	$\gamma_3 = 32\exp(w_7)$
$\delta_{21} = 16\exp(w_4)$	$\gamma_4 = 32\exp(w_8)$
$r_1 = \frac{2}{3}\exp(w_9)$	$r_2 = \frac{1}{3}\exp(w_{10})$

Transformation from model parameters, w, to physiological variables in Neural Mass Models.
