# Supplementary material for: Annealed Importance Sampling for Neural Mass Models
Source: PLoS Comput Biol. 2016 Mar 4;12(3):e1004797. doi: 10.1371/journal.pcbi.1004797 (PMC4778905; doi:10.1371/journal.pcbi.1004797)
Supplement: S2 Table — (PDF) [file pcbi.1004797.s012.pdf]

Table 1. Gaussianity Test for data from Full Neural Mass Model

SNR	32 Trajectories		64 Trajectories	
	Full	Reduced	Full	Reduced
1	0.02	0.18	0.02	0.03
2	0.40	0.10	0.74	0.42
4	0.80	0.54	0.07	0.29
8	0.33	0.51	0.004	0.02
16	0.40	0.17	0.02	5×10^{-4}

p-values from Royston's Gaussianity test applied to AIS samples. The fitted model is either Full or Reduced.
