# Supplementary material for: Annealed Importance Sampling for Neural Mass Models
Source: PLoS Comput Biol. 2016 Mar 4;12(3):e1004797. doi: 10.1371/journal.pcbi.1004797 (PMC4778905; doi:10.1371/journal.pcbi.1004797)
Supplement: S3 Table — (PDF) [file pcbi.1004797.s013.pdf]

Table 1. Gaussianity Test for data from Reduced Neural Mass Model

SNR	32 Trajectories		64 Trajectories	
	Full	Reduced	Full	Reduced
1	0.04	0.60	0.32	0.05
2	0.86	0.87	0.55	0.54
4	0.40	0.72	0.46	0.49
8	0.02	0.52	0.57	0.40
16	0.11	0.15	0.08	0.11

p-values from Royston's Gaussianity test applied to AIS samples. The fitted model is either Full or Reduced.
